# Insight into the outer membrane asymmetry of *P. aeruginosa* and the role of MlaA in modulating the lipidic composition, mechanical, biophysical, and functional membrane properties of the cell envelope

**DOI:** 10.1128/spectrum.01484-24

**Published:** 2024-10-07

**Authors:** M. Kaur, N. Mozaheb, T. O. Paiva, M.-F. Herent, F. Goormaghtigh, A. Paquot, R. Terrasi, E. Mignolet, J.-L. Décout, J. H. Lorent, Y. Larondelle, G. G. Muccioli, J. Quetin-Leclercq, Y. F. Dufrêne, M.-P. Mingeot-Leclercq

**Affiliations:** 1 UCLouvain, Louvain Drug Research Institute, Cellular & Molecular Pharmacology, Brussels, Belgium; 2 UCLouvain, Louvain Institute of Biomolecular Science and Technology, nanoBiophysics, Louvain-la-Neuve, Belgium; 3 UCLouvain, Louvain Drug Research Institute, Pharmacognosy, Brussels, Belgium; 4 UCLouvain, Louvain Drug Research Institute, Bioanalysis and Pharmacology of Bioactive Lipids, Brussels, Belgium; 5 UCLouvain, Louvain Institute of Biomolecular Science and Technology, Biochemistry of Nutrition and Environmental Toxicology Louvain-la-Neuve, Brussels, Belgium; 6 Université Grenoble Alpes, CNRS, DPM, Grenoble, France; Centre National de la Recherche Scientifique, Marseille, France

**Keywords:** *P. aeruginosa*, outer membrane, membrane biophysics, lipids, cell envelope

## Abstract

In Gram-negative bacteria, the outer membrane (OM) is asymmetric, with lipopolysaccharides (LPS) in the outer leaflet and glycerophospholipids (GPLs) in the inner leaflet. The asymmetry is maintained by the Mla system (MlaA-MlaBCDEF), which contributes to lipid homeostasis by removing mislocalized GPLs from the outer leaflet of the OM. Here, we ascribed how *Pseudomonas aeruginosa* ATCC 27853 coordinately regulates pathways to provide defense against the threats posed by the deletion of *mlaA*. Especially, we explored (i) the effects on membrane lipid composition including LPS, GPLs, and lysophospholipids, (ii) the biophysical properties of the OM such as stiffness and fluidity, and (iii) the impact of these changes on permeability, antibiotic susceptibility, and membrane vesicles (MVs) generation. Deletion of *mlaA* induced an increase in total GPLs and a decrease in LPS level while also triggering alterations in lipid A structures (arabinosylation and palmitoylation), likely to be induced by a two-component system (PhoPQ-PmrAB). Altered lipid composition may serve a physiological purpose in regulating the mechanobiological and functional properties of *P. aerugino*sa. We demonstrated an increase in cell stiffness without alteration of turgor pressure and inner membrane (IM) fluidity in ∆*mlaA*. In addition, membrane vesiculation increased without any change in OM/IM permeability. An amphiphilic aminoglycoside derivative (3’,6-dinonyl neamine) that targets *P. aeruginosa* membranes induced an opposite effect on ∆*mlaA* strain with a trend toward a return to the situation observed for the WT strain. Efforts dedicated to understanding the crosstalk between the OM lipid composition, and the mechanical behavior of bacterial envelope, is one needed step for designing new targets or new drugs to fight *P. aeruginosa* infections.

**IMPORTANCE:**

*Pseudomonas aeruginosa* is a Gram-negative bacterium responsible for severe hospital-acquired infections. The outer membrane (OM) of Gram-negative bacteria acts as an effective barrier against toxic compounds, and therefore, compromising this structure could increase sensitivity to antibiotics. The OM is asymmetric with the highly packed lipopolysaccharide monolayer at the outer leaflet and glycerophospholipids at the inner leaflet. OM asymmetry is maintained by the Mla pathway resulting in the retrograde transport of glycerophospholipids from the OM to the inner membrane. In this study, we show that deleting *mlaA*, the membrane component of Mla system located at the OM, affects the mechanical and functional properties of *P. aeruginosa* cell envelope. Our results provide insights into the role of MlaA, involved in the Mla transport pathway in *P. aeruginosa*.

## INTRODUCTION

In Gram-negative bacteria, like *Pseudomonas aeruginosa*, the cell envelope is made up of an inner phospholipid bilayer (IM), a periplasm that contains a thin peptidoglycan cell wall and an outer membrane (OM) presenting an asymmetric distribution of lipids on both leaflets. The outer leaflet is almost exclusively composed of lipopolysaccharides (LPS), whereas the inner leaflet mainly contains glycerophospholipids (GPLs). LPS is a negatively-charged amphiphilic molecule composed of three covalently linked moieties: lipid A, a core oligosaccharide, and a polysaccharide called O-antigen. GPLs are mainly phosphatidylethanolamine (PE), phosphatidylglycerol (PG), and cardiolipin (CL), which are also found in the inner membrane (1).

The asymmetric distribution of LPS and GPLs in the OM is critical for maintaining membrane mechanical properties required for cell fitness and protection against environmental stress and antibiotics. This is elucidated by the fact that a loss of OM asymmetry is responsible for a decrease in permeability barrier function (2, 3), a reduction in virulence (4–7), and the dwindling of intrinsic resistance to some antimicrobials (8–10).

The maintenance of lipid asymmetry is therefore crucial and is in part controlled by the Mla (MlaA/VacJ-MlaC-MlaFEDB) system. The Mla system, composed of 6 proteins, facilitates retrograde phospholipid transport from the OM back to the IM (11). The MlaA (also annotated as VacJ) component is highly conserved among Gram-negative bacteria (Table 1). It was first identified in *Shigella flexeneri* as virulence-associated protein chromosome locus J (6). It is an integral alpha-helical OM protein (12) that transfers phospholipids from the outer leaflet of OM to the soluble periplasmic MlaC with no preference for specific phospholipids (13). MlaC (14) transfers the phospholipids to the multicomponent system MlaFEDB (13, 15). This ATP-binding-cassette (ABC) transport system in the IM comprises the IM permease MlaE, the IM ABC-type ATPase MlaF, the periplasmic subunit of MlaFEDB MlaD, which is anchored in the IM by a transmembrane helix, and a cytoplasmic accessory protein MlaB (13, 16).

**TABLE 1:** MlaA amino acids sequence conservation in *P. aeruginosa* and other Gram-negative bacteria

Microorganisms	Accession number	MlaA/VacJ	Amino acids
*P. aeruginosa*	AVK06939.1	100%	234
*Pseudomonas putida*	AFO47095.1	69.36%	235
*Pseudomonas syringae*	QWB08392.1	66.67%	233
*Acinetobacter baumannii*	BCR39766.1	41.75%	299
*Neisseria gonorrhoeae*	WP_010951410.1	38.39%	277
*Campylobacter jejuni*	EDP6655880.1	36.65%	232
*Caulobacter crescentus*	QXZ52028.1	36.70%	286
*Vibrio cholerae*	ACP06275.1	35.84%	254
*Haemophilus influenzae*	AIT66950.1	33.82%	250
*Shigella flexneri* 2 a	NP_708228.1	33.04%	251
*Escherichia coli*	QKU50363.1	33.04%	251
*Salmonella enterica typhimurium*	WP_000776787.1	32.17%	251

In addition to the Mla system, lipid asymmetry in Gram-negative bacteria is maintained by the OM-phospholipase PldA (17, 18) and the palmitoyl transferase PagP (19). PldA cleaves GPLs at the *sn*-1 or *sn*-2 position, generating fatty acids and lysophospholipids (20), which can flip to the inner leaflet of the OM, where they are trafficked back to the IM by poorly understood mechanisms. Phospholipids at the inner leaflet of the OM are typically inaccessible to the active site of PldA (17, 21, 22). Disturbance of the OM integrity and asymmetry leads to the activation of PldA via a Ca^2+^-dependent dimerization mechanism (22, 23). PagP regulates lipid asymmetry of the OM by transferring *sn*-1 palmitate (C:16) from GPLs at the inner leaflet to lipid A at the outer leaflet, producing palmitoylated lipid A (11). Palmitoylated lipid A increases membrane hydrophobicity (24) and activates the periplasmic σ^E^ stress response (25). PagP activity is an established marker for monitoring ectopic GPL exposure in the OM of *E. coli* (26, 27) and accumulation of GPLs in the outer leaflet of the OM in cells lacking MlaA (4, 11). On *Klebsiella pneumoniae* (24) and *Salmonella typhimurium* (28), PagP showed a bifunctional activity with additional capacity to transfer a palmitoyl moiety to PG, which also increases membrane hydrophobicity (24) and promotes the generation of membrane vesicles (MVs) (28).

Regulating cell wall properties, including lipid asymmetry, plays a key role in antibiotic resistance and bacterial survival and thus might provide a source for antibiotic targets. Since the OM is the first place where antibiotics interact with the bacteria, we selected to focus on the protein from the Mla system, located at the OM, MlaA. On *P. aeruginosa* WT and ∆*mlaA*, we characterized the lipidic composition, the mechanical and biophysical properties of the cell envelope, and the significance of changes induced by *mlaA* deletion. Especially, we explored the effects of *mlaA* deletion on (i) the lipid composition of membranes including LPS, GPLs, and lysophospholipids, (ii) the biophysical properties of the OM (stiffness, fluidity, etc.), and (iii) the impact of these changes on permeability, antibiotic susceptibility, and MVs generation. All studies were conducted in the presence and absence of the amphiphilic aminoglycoside derivative, 3',6-dinonyl neamine (3’,6-diNn), known to bind to the main components of the OM, LPS (29), and GPLs (30, 31).

## RESULTS

### Characterization of *P. aeruginosa* ∆*mlaA* strain

Gene-specific PCR followed by electrophoresis was performed on DNA extracted from *P. aeruginosa* WT and ∆*mlaA*. We confirmed the deletion of *mlaA* (Fig. S1C). The mRNA expression of other *mla* genes (*mlaB*, *mlaC*, *mlaD*, *mlaE,* and *mlaF*) in ∆*mla* over WT strain showed an increase from 1.3-fold to 2.6-fold (Fig. S1D). *P. aeruginosa* strains, WT, ∆*mlaA*, and ∆*mla att7::mlaA* did not show any differences in growth curves, intracellular survival, and morphology (5).

### Deletion of *mlaA* induced an increase in glycerophospholipids (GPLs) at the OM of *P. aeruginosa*. 3’,6-dinonyl neamine increased GPLs in the WT strain but not in *P. aeruginosa* ∆*mlaA*

The Mla system maintains OM-lipid asymmetry and traffics GPLs between the OM and IM bilayers (11, 32, 33). We hypothesized that *mlaA* deletion induced altered OM lipid composition. Therefore, we determined the total amounts of GPLs in the OM of *P. aeruginosa* WT and ∆*mlaA*. We then investigated the changes in individual GPLs, including phosphatidylethanolamine (PE), phosphatidylglycerol (PG), and cardiolipin (CL). To assess the potential role of PldA and/or PagP, we monitored the amounts of lyso-derivatives (lysophosphatidylethanolamine, LPE; lysophosphatidylglycerol, LPG). The degree of unsaturation was determined as well.

**Fig 1:**
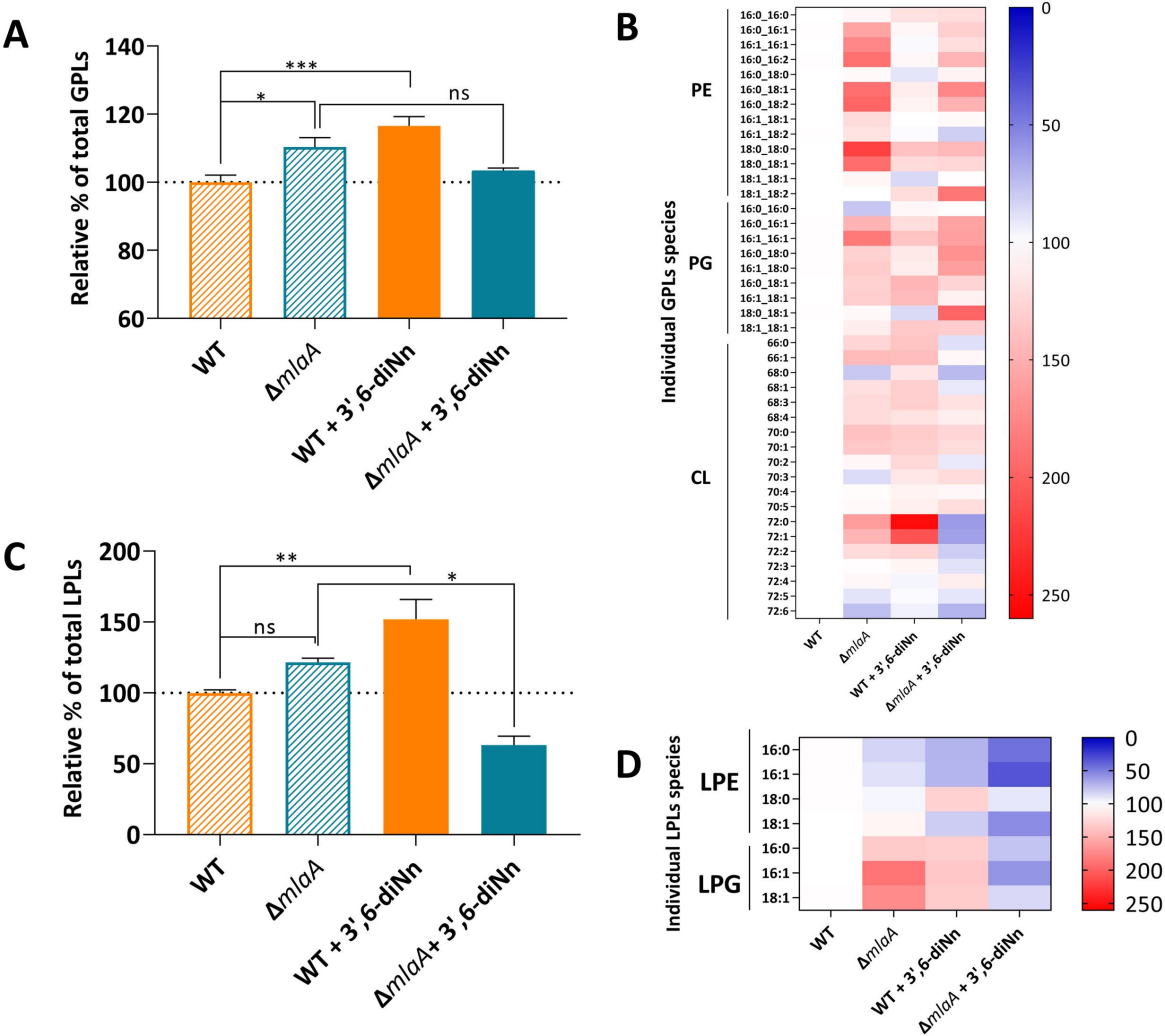
Glycerophospholipids and lysophospholipids profile *P. aeruginosa* WT and ∆*mlaA* in the presence or absence of 3’,6-dinonyl neamine (1× MIC, 2 µg/mL, 1 h). (**A and C**) Total glycerophospholipids. (**B and D**) Heat maps of individual glycerophospholipids and lysophosphoglycerides species. Red-filled, blue-filled, and white-filled lines referred to increased, decreased, and unchanged levels of lipid structures, respectively. Total lipids were determined by using a fluorescent probe FM4-64, and individual lipids PE, PG, CL, LPE, and LPG were analyzed by LC-MS. The results are expressed in % where WT is considered 100%. Statistical analysis was performed using one-way ANOVA with Tukey’s multiple-comparison test. (**A and C**) ***P < 0.001, **P < 0.01, *P < 0.05; ^ns^ *P* > 0.05.

Focusing on total GPLs, we showed that deletion of *mla*A (∆*mlaA*) increased total GPLs in *P. aeruginosa* OM (110% ± 6%) in comparison with *P. aeruginosa* WT (Fig. 1A). A deeper analysis of individual lipid headgroups (PE, PG, and CL) (Fig. 1B) revealed a significant increase in PE in ∆*mlaA* strain compared with WT bacteria (Fig. S3A). The increase was significant for the PE species (16:0_16:1, 16:0_16:2, 16:0_18:1, and 18:1_18:2) (Fig. S2A).

The addition of 3’,6-dinonyl neamine on *P. aeruginosa* WT induced a significant increase in GPLs (117% ± 7%), whereas no significant change was observed when adding the compound to *P. aeruginosa* ∆*mlaA* (Fig. 1A). In WT strain, an analysis on the individual GPL species showed a significant increase in one CL species (72:0) (Fig. S2C) without any significant global change for PE, PG, and CL (Fig. S3A). In *P. aeruginosa* ∆*mlaA,* incubation with 3’,6-dinonyl neamine did not alter the glycerophospholipids species (Fig. S2A through C and S3A).

The presence of higher amounts of GPLs at the OM of *P. aeruginosa* ∆*mlaA* might activate enzymes like PldA and/or PagP, resulting in the production of lyso-derivatives (34). Lysophospholipids are the products of GPLs hydrolysis by endogenous PldA or by-products of PagP. Interestingly, the profile of total lyso-derivatives was similar to that observed for GPLs with a tendency that lyso-derivatives in OM of *P. aeruginosa* ∆*mlaA* strain increased (122% ± 4%) when compared with WT (100% ± 4%) (Fig. 1C). At the headgroup level (Fig. 1D), the increase mostly resulted from LPG compared with LPE (Fig. S2D and S3B). As for GPLs, the addition of 3’,6-dinonyl neamine resulted in an increase in total lyso-derivatives in WT (152% ± 20%) (Fig. 1C) resulting from the increase in LPG (Fig. S3B). In *P. aeruginosa* ∆*mlaA,* a huge decrease (66% ± 8%) in lyso-derivatives was observed upon the addition of the compound (Fig. 1C), which affected both LPE and LPG (Fig. S3B).

Comparing lipid acyl chains, we observed that monounsaturated and polyunsaturated acyl chains increased in *P. aeruginosa* ∆*mlaA* compared with WT (Fig. 1B; Fig. S3C). Addition of 3’,6-dinonyl neamine on *P. aeruginosa* WT increased the three subtypes of acylated chains with a significant effect observed for saturated chains (Fig. 1B and D; Fig. S2A through D and S3C), whereas no significant change was observed for ∆*mlaA* strain (Fig. 1B; Fig. S2A through D and S3C).

In conclusion, the deletion of *mlaA* induced an increase in the amounts of GPLs at the OM of *P. aeruginosa* with a major effect on PE. 3’,6-dinonyl neamine increased GPLs in the WT strain without any significant changes in *P. aeruginosa* ∆*mlaA*.

### Deletion of *mla*A in *P. aeruginosa* induced a decrease in LPS and a increase in lipid A modifications by increasing palmitoylation and arabinosylation. Higher expression of two two-component systems, PhoPQ and PmrAB, is observed as well

**Fig 2:**
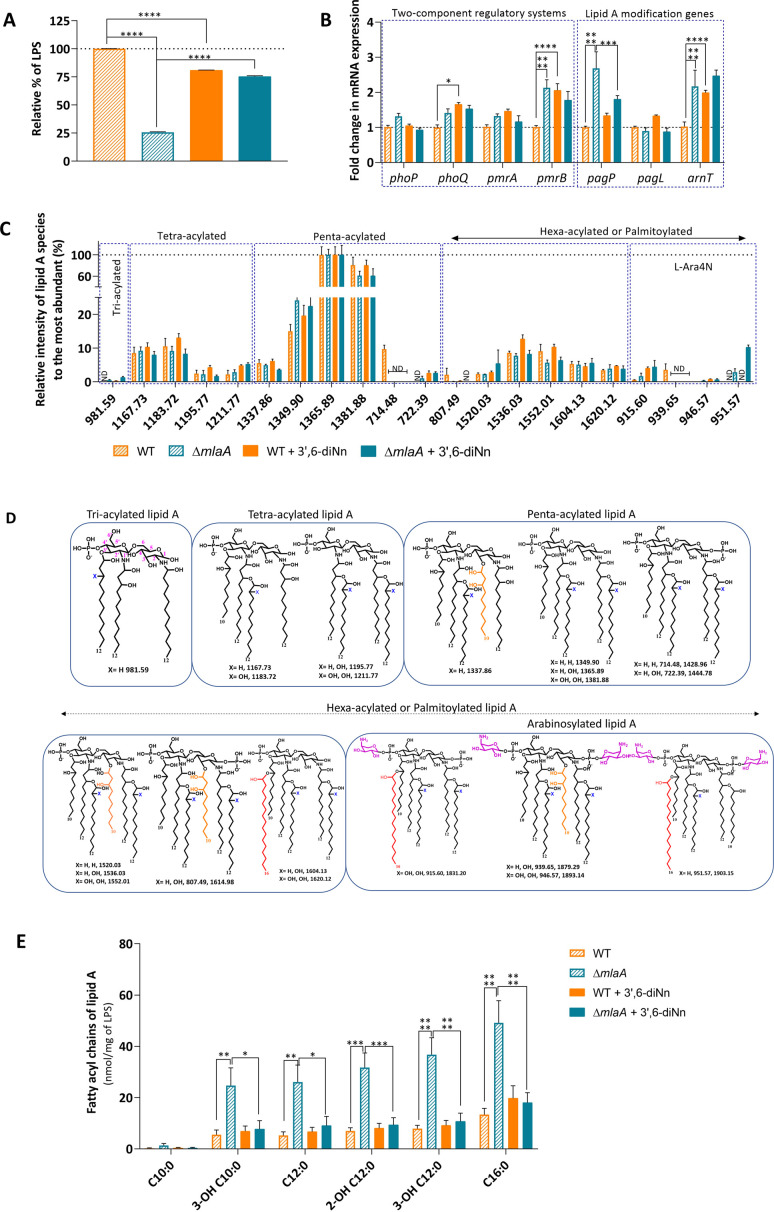
Lipopolysaccharides (LPS) and lipid A modifications in *P. aeruginosa* WT and ∆*mlaA* non-treated and treated with 3’,6-dinonyl neamine (1× MIC, 2 µg/mL, 1 h). (**A**) Total LPS was measured by purpald assay. The results are expressed in relative % over *P. aeruginosa* WT, considered 100% (13,444 ± 16 µg/mL). (**B**) mRNA expression of two-component regulatory systems (*phoP*, *phoQ, pmrA,* and *pmrB,*) and lipid A modifications genes (*pagP, pagL,* and *arnT*). The results are expressed in fold change over *P. aeruginosa* WT. (**C**) MS peaks (*m/z*) obtained by ESI-MS (negative ion mode) shown in different boxes as tri-acylated, tetra-acylated, penta-acylated, hexa-acylated, palmitoylated, and arabinosylated with their relative intensity compared with the major peak for each condition (*m/z* 1365.89). (**D**) Predicted structures of lipid A structures from *m/z* were obtained. The positions of the acyl chain are hypothetical; 4-aminoarabinose added by ArnT is shown in purple, and palmitate chain added by PagP is shown in red on lipid A structures. (**E**) Fatty acids were isolated from lipid A structures and analyzed as FAMEs using gas chromatography - mass spectrometry (GC-MS). Statistical analysis was performed by two-way ANOVA with multiple comparisons (**B, C, E**) and one-way ANOVA with Tukey’s multiple-comparison test (**A**) ****P < 0.0001, ***P < 0.001, *P < 0.05, ND undetectable. All values are given in mean ± SEM, and the experiment was repeated thrice.

Perturbing OM asymmetry results in signal transduction across the periplasm and a lipid-mediated regulatory mechanism that controls LPS biosynthesis (17, 35). Accordingly, we hypothesized that *mlaA* deletion might influence the LPS levels in *P. aeruginosa*. Relative to the WT strain, the LPS levels of the Δ*mlaA* strain were significantly decreased (by 75%) (Fig. 2), similar to what was reported for *∆mlaF* mutant of *A. baumannii* (36). The effect of 3’,6-dinonyl neamine is dependent upon the presence of MlaA, with a decrease in WT (by 19%) and a huge increase in ∆*mlaA* (Fig. 2A) strain (293%).

To know whether the decrease in LPS content in *P. aeruginosa* ∆*mlaA* is accompanied by structural modifications of the LPS moiety embedded in lipids (lipid A), we explored the activation of two two-component systems (TCSs), PhoP-PhoQ and PmrA-PmrB (37). In these systems, a sensory kinase, that is, PhoQ or PmrB, is located at the IM and is activated by an environmental signal, which in turn phosphorylates a cytoplasmic transcriptional regulator, PhoP or PmrA, respectively. PhoPQ and PmrAB are involved in OM remodeling by lipid A modifications in LPS through palmitoylation *via* PagP, which transfers a palmitoyl group from a GPL to the β-OH of the 3′-O-acyl fatty acyl group, deacylation *via* PagL, which removes 3-OH C:10, arabinosylation *via* ArnT, which adds 4-amino-4-deoxy-L-arabinose to one or both of the 1 and 4’-phosphate groups, and/or hydroxylation of secondary acyl chains via LpxO1 and LpxO2.

Deletion of *mla*A (∆*mlaA*) led to the increased expression of the *pmrB* (2.1-fold), as well as the TCSs-induced lipid A modification genes (*pagP* 2.7-fold and *arnT* 2.2-fold) without any effect on *pmrA, phoP, phoQ,* and *pagL* (Fig. 2B). Upregulation of *pagP*, and *arnT* also reported in *Salmonella enterica* subsp. *enterica* serovar *Typhimurium,* helps to stabilize the OM, with an ultimate decrease in membrane fluidity and an increase in the surface charge (38, 39). Exposure of *P. aeruginosa* WT to 3’,6-dinonyl neamine induced an increase in the expression of *pmrB* (2.1-fold), *phoQ* (1.7-fold), and *arnT* (2.2-fold) (Fig. 2B). In the ∆*mlaA* strain, 3’,6-dinonyl neamine exposure decreased the expression of *pagP* (by 33%) (Fig. 2B).

To further assess changes in lipid A, we used high-resolution mass spectrometry. Overall, the mass spectrum of lipid A characterized 14 single-charged [M-H]^-^ and seven double-charged [M-2H]^2-^ lipid A structures that were tri-, tetra-, penta-, or hexa-acylated. Among these, some were palmitoylated and/or arabinosylated (Fig. 2C and D; Tables S2 and S3). In *P. aeruginosa* WT and ∆*mlaA*, before as well as after the treatment with 3’,6-dinonyl neamine, the most intense ion was *m/z* of 1365.89 (PLA1), which represents a mono-phosphate penta-acylated lipid A form (see detailed results in the supplementary part). Furthermore, we determined the levels of lipid A acyl chains after normalization to total LPS *via* derivatization/gas chromatography (Fatty Acid Methyl Esters; FAMEs) (Fig. 2A). We observed an increase in 2-OH C12:0, 3-OH C12:0 in *P. aeruginosa* ∆*mlaA* compared with WT strain. How these changes are related to the activity of LpxO1/2 known to α-hydroxylate the 2’- and 2- secondary acyl chains of lipid A (40) is unclear. C16:0 also increased in the *P. aeruginosa* ∆*mlaA* but decreased upon treatment with 3’,6-dinonyl neamine (Fig. 2E), consistent with the expression of *pagP* (Fig. 2B).

In summary, the absence of *mlaA* induced lipid A modifications through potential activation of PhoPQ and PmrAB TCSs, which in turn induced lipid A palmitoylation and arabinosylation. 3’,6-dinonyl neamine also induced lipid A modifications through activation of PhoPQ and PmrAB, increasing mRNA expression of *pagP*, *pagL,* and *arnT* in *P. aeruginosa* WT, whereas a decrease in mRNA expression of *pagP* was observed in ∆*mlaA* strain. The increase of arabinosylation is in line with the increase in zeta potential (−31.8 mV in ∆*mlaA* strain) compared with the WT strain (−37.6 mV) (Fig. S5). If and how these changes in OM lipid composition affect the mechanobiological properties of OM was the next question we addressed by using atomic force microscopy (AFM).

### Deletion of *mlaA* induced an increase in the stiffness of the cell envelope without altering the turgor pressure. 3’,6-dinonyl neamine induced a higher softening of the cell envelope of the *P. aeruginosa* Δ*mlaA*

**Fig 3:**
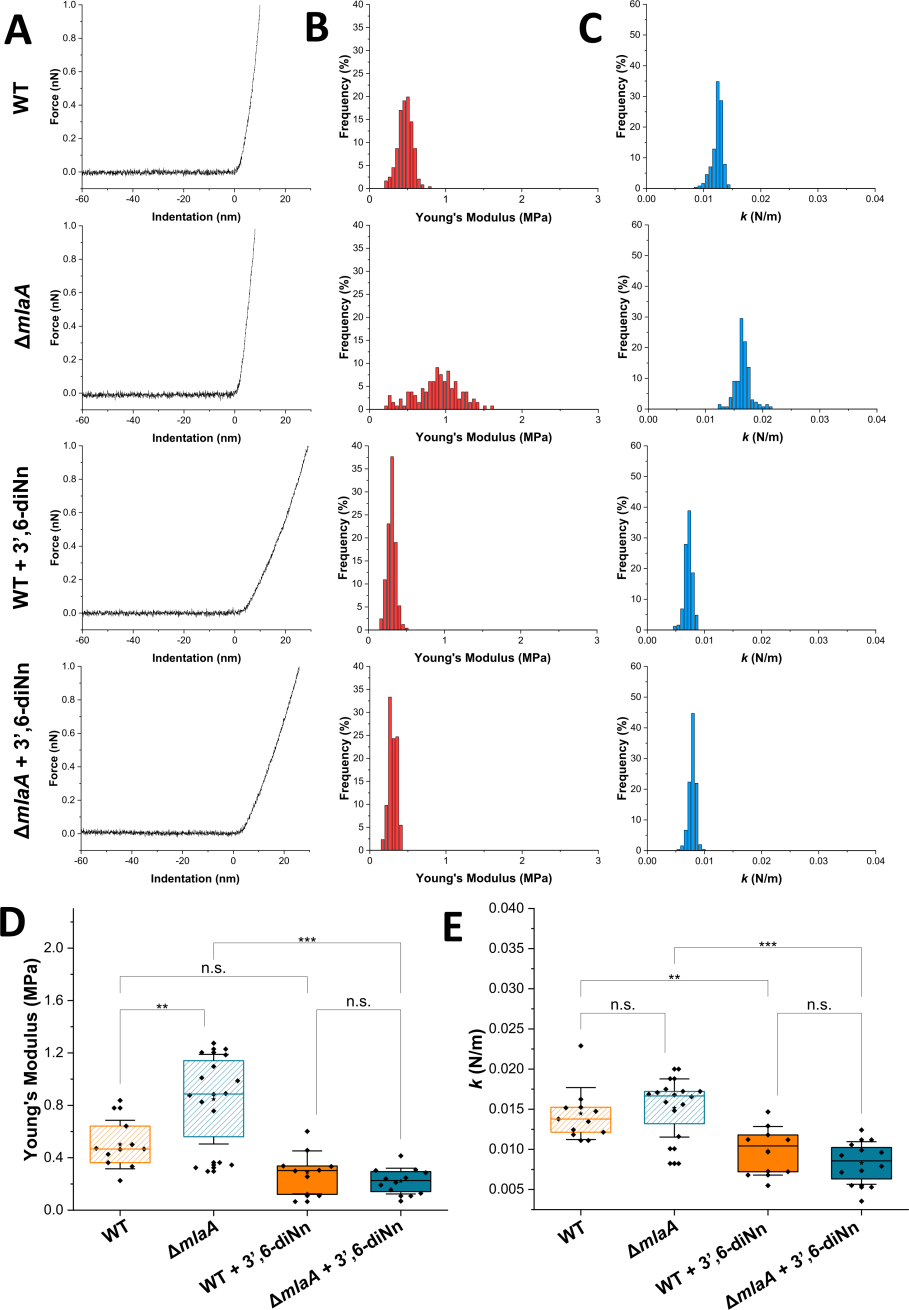
Characterization of the cell envelope mechanics by AFM. (**A**) Representative force-indentation curves and distributions of (**B**) Young’s modulus and (**C**) spring constant of one representative *P. aeruginosa* cell of WT and Δ*mla*A strains, before and after treatment with 3’,6-dinonyl neamine (1× MIC, 2 µg/L, 1 h). (**D**) Box plot shows the Young’s modulus, and (**E**) spring constant values were calculated from the force-indentation curves for both strains, before and after antibiotic treatment. (**D and E**) Statistical analyses were carried out using a statistical Tukey’s multiple-comparison test. Stars indicate the mean values, lines the medians, boxes indicate the 25%–75% quartiles, and whiskers indicate the standard deviation obtained from at least 10 independent cells over at least three independent experiments ***P ≤ 0.001, **P ≤ 0.01, and ^ns^ *P* > 0.01, determined by Tukey’s multiple-comparison test.

AFM has emerged as a valuable multifunctional technique to investigate the structure and mechanical properties of microbial cells. In mechanobiology, AFM-based indentation experiments have enabled to quantify the mechanical properties of whole bacterial cells and isolated cell walls (41). Spatially resolved force-distance curves were collected on top of the bacteria (Fig. 3A), and respective elasticity (Fig. 3B) and stiffness (Fig. 3C) information was extracted from them (41, 42). Representative force-indentation curves are shown in Fig. 3A, for both *P. aeruginosa* WT and Δ*mlaA* strains before and after treatment with 3’,6-dinonyl neamine at the minimum inhibitory concentration (MIC) for 1 h. As expected for Gram-negative bacteria, the curves featured two regions, that is, a nonlinear regime at low forces reflecting cell wall elasticity, followed by a linear regime at high forces associated with turgor pressure. From these two regions, we calculated the bacterial Young’s modulus, *E*, and the bacterial spring constant, *k* (43, 44). Representative distributions of *E* and *k* values are shown in Fig. 3B and C, respectively.

A Young’s modulus of 0.50 ± 0.18 MPa (mean ± SD, *n* = 11 cells) was measured for *P. aeruginosa* WT (Fig. 3D), a value higher than that of 0.24 MPa ± 0.04 published by Formosa et al (45), a difference that we attribute to intercellular variability and differences in experimental conditions.

Interestingly, the Young’s modulus value here calculated for *P. aeruginosa* WT was slightly lower than those published for *E. coli* (ranging from 1 to a few MPa) (46–48). This difference can be explained by (i) the thinner peptidoglycan layer of *P. aeruginosa* compared with *E. coli* (49), (ii) a reduced interaction between peptidoglycan and the OM (50) and, (iii) differences in lipid composition as lower amounts of very long chain fatty acids covalently bonded to hopanoids (51) or the shorter fatty acyl chains of lipid A (C12) from *P. aeruginosa* compared with other Gram-negative bacteria including *A. baumannii* (C12-C14) and *Burkholderia mallei* (C14-C16) (52). Deletion of *mlaA* significantly increased Young’s modulus (Fig. 3D) as it reached 0.84 ± 0.34 MPa (*n* = 16 cells), indicating a stiffening of the cell envelope.

A decrease in Young’s modulus (Fig. 3D) to 0.29 ± 0.164 MPa (*n* = 10 cells) and 0.22 ± 0.10 MPa (*n* = 12 cells) was observed upon incubation with 3’,6-dinonyl neamine, for the WT and ∆*mlaA* strains, respectively.

The *k* values (Fig. 3E) were found to be similar for WT and *mlaA* deleted strains (0.015 ± 0.003 and 0.015 ± 0.004 N/m, *n* = 11, 16 cells), indicating similar levels of turgor pressure. These values are in the range of those reported by Yao et al for *P. aeruginosa* (0.02  N/m) (49) as well as for *Shewanella putrefaciens* (0.02–0.05  N/m), another Gram-negative bacterium (53). The spring constants here calculated for *P. aeruginosa* were lower compared with *E. coli* (0.04  N/m) (54, 55). Treatment with 3’,6-dinonyl neamine resulted in a decrease of *k* to 0.010 ± 0.003 N/m (WT, *n* = 10 cells) and 0.008 ± 0.003 N/m (Δ*mlaA*, *n* = 12 cells), indicating a turgor pressure decrease as a result of the antibiotic action.

**Fig 4:**
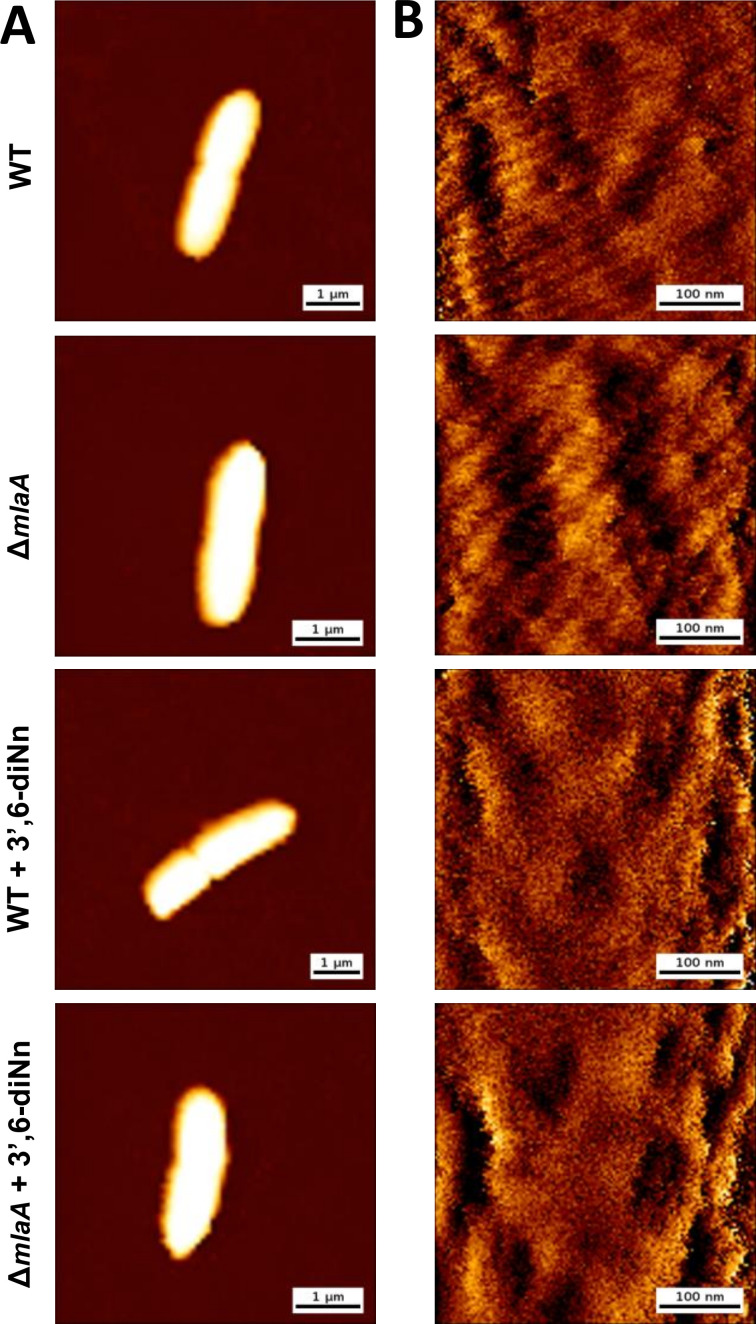
AFM topographical characterization of *P. aeruginosa* WT and ∆*mlaA* non-treated and treated with 3’,6-dinonyl neamine (1× MIC, 2 mg/L, 1 h). (**A**) Representative AFM height images of *P. aeruginosa* WT and Δ*mlaA* strains, before and after treatment with antibiotic 3’,6-dinonyl neamine (1× MIC, 2 µg/mL, 1 h). Color scale: 650 nm. (**B**) High-resolution height images were recorded on top of the cells. Color scale: 20 nm. The images were recorded in phosphate-buffered saline (PBS), and the cells were immobilized on glass-bottom dishes previously functionalized with poly-L-lysine.

In parallel, AFM topographical characterization of the cell morphology was performed in quantitative imaging mode (Fig. 4). As can be seen in Fig. 4A, both WT and Δ*mlaA* strains showed the characteristic rod shape of *P. aeruginosa* cells. No morphological differences were observed between the two strains, as confirmed by the high-resolution images recorded on top of the cells (Fig. 4B). Similar morphological features were observed upon treatment with 3’,6-dinonyl neamine, with no significative changes detected on the surface ultrastructure as evaluated by AFM. Identical height values, extracted from the AFM images, were observed for *P. aeruginosa* cells from both before and after antibiotic treatment (Fig. S5). Importantly, this observation validates the use of AFM in the present study, excluding the role of possible AFM artifacts/interferences to the conclusions described above.

### Deletion of *mlaA* did not change IM fluidity in *P. aeruginosa*. 3’,6-dinonyl neamine increased fluidity of IM in WT without any effect on ∆*mlaA* strain

**Fig 5:**
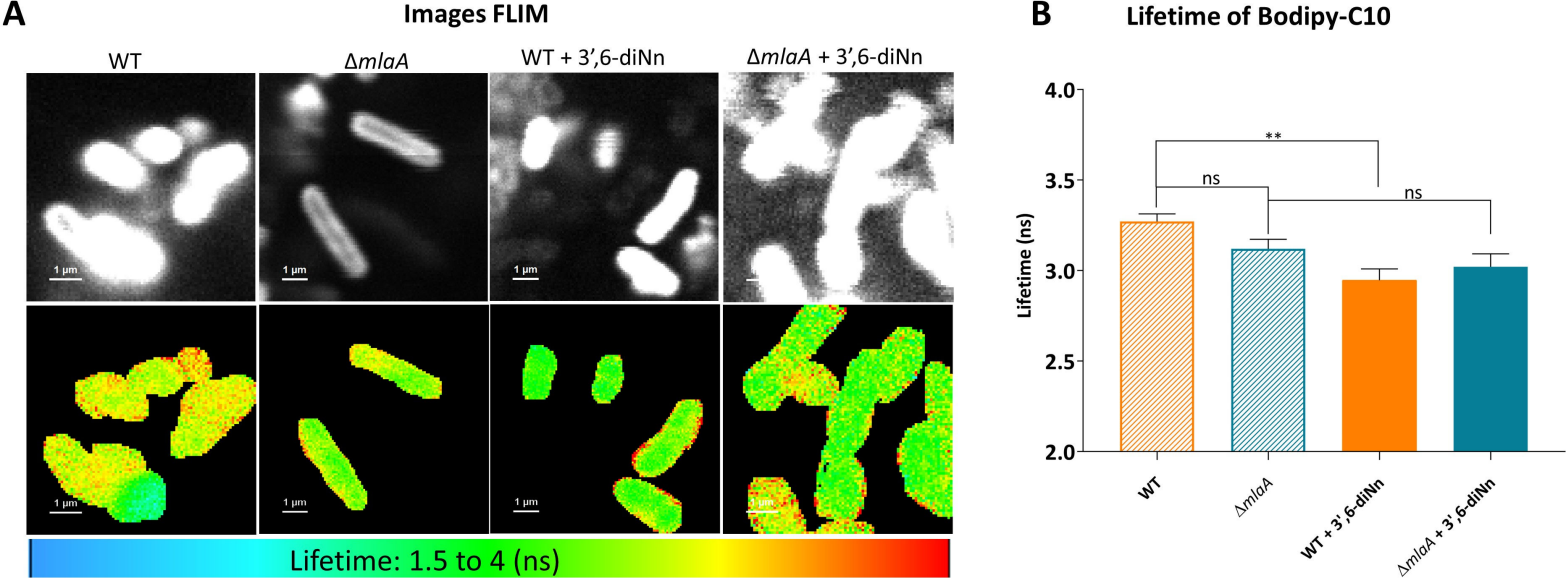
FLIM images with their lifetime. *P. aeruginosa* WT and ∆*mlaA* in the presence and absence of 3’,6 dinonyl neamine (1× MIC, 2 mg/L, 1 h). (**A**) Top panels are intensity images, and the below panels are pseudo-colored FLIM images. (**B**) Lifetime measurements of the bacteria stained with BODIPY-C10. The data were obtained from two individual experiments, and in each experiment, at least 10 bacteria or three slides were considered to measure the lifetimes. The data are presented as means ± SD from three independent replicates. Statistical analysis was performed by one-way ANOVA with Tukey’s multiple-comparison test. **P < 0.01 and ^ns^ *P* >0.05.

Since the mechanical parameters of the cell envelope were changed, we were wondering if alterations of membrane biophysical parameters such as IM fluidity would also be affected. By using fluorescence-lifetime imaging microscopy (FLIM), we monitored the fluorescence lifetime of BODIPY-C10, which correlates with the viscosity of the environment in which the probe is embedded (56). In our analysis, a longer fluorescence lifetime of the probe indicates a higher viscosity or a lower fluidity of the membrane (57). The FLIM analysis showed that membrane fluidity of ∆*mlaA* is similar to that of WT (Fig. 5A and B). 3’,6-dinonyl increased fluidity in *P. aeruginosa* WT without any significant effect on *P. aeruginosa* ∆*mlaA* (Fig. 5B).

### Deletion of *mlaA* did not change OM and IM permeability. *P. aeruginosa* ∆*mlaA* was less sensitive to OM permeabilization induced by 3’,6 dinonyl neamine compared with WT, whereas no difference was observed in IM

**Fig 6:**
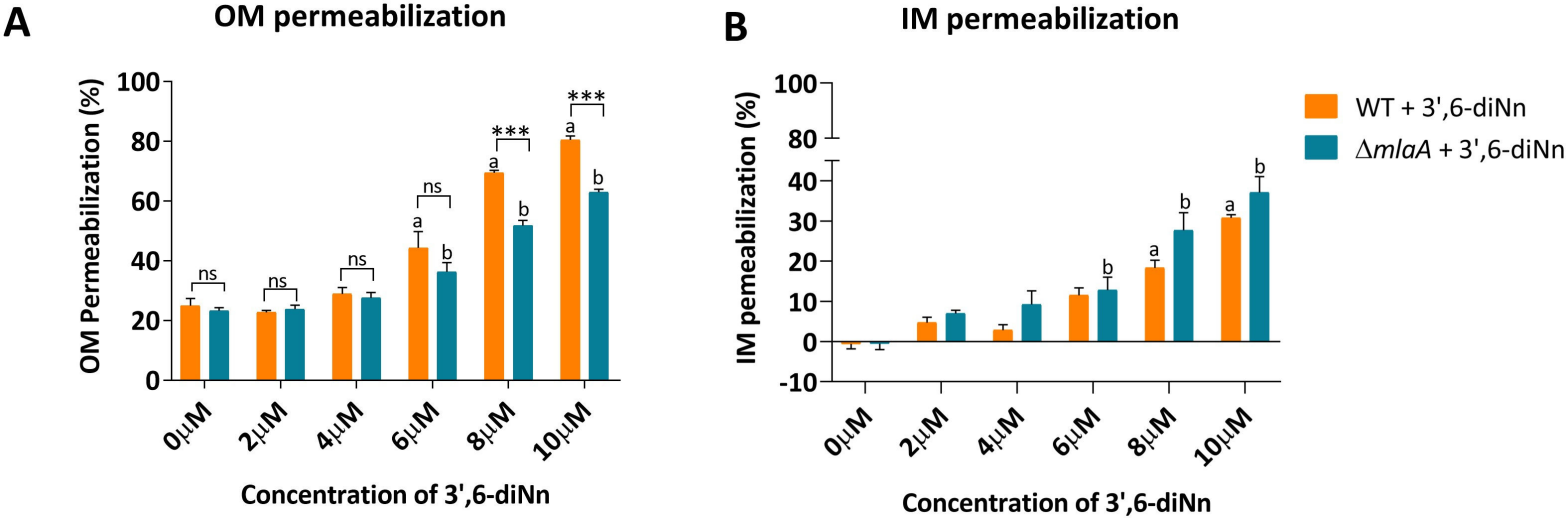
Membrane permeabilization. (**A**) Outer membrane permeabilization was assessed by nitrocefin assay (**B**). Inner membrane permeabilization was assessed by propidium iodide assay where WT (orange) and ∆*mlaA* (blue) were incubated with different concentrations (0–10 µM) of 3’,6-dinonyl neamine. Statistical analysis was performed using two-way ANOVA with multiple comparisons. ***P < 0.001; ^ns^ *P* >0.05; (**A and B**) significant increase in membrane permeabilization compared with untreated *P. aeruginosa* WT (**A**) and ∆*mlaA* (**B**).

The amphipathic nature of LPS contributes to the permeability barrier of the OM (3). Changes in LPS contents and structural variations in lipid A may increase or decrease the permeation of hydrophobic compounds (58). We first determined OM and IM permeability in *P. aeruginosa* WT and ∆*mlaA*. On both strains, we also evaluated the effect of 3’,6-dinonyl neamine known to interact with LPS in the OM of *P. aeruginosa* (29). The OM permeability (Fig. 6A) was determined by using nitrocefin, a chromogenic β-lactam compound that undergoes a color change from yellow to red upon hydrolysis. In whole-cell Gram-negative bacteria, β-lactamases are primarily located in the periplasm; therefore, the nitrocefin hydrolysis rate is primarily limited by its diffusion rate across the OM (59). In the absence of antibiotics, both strains did not show any difference in OM permeability (10), suggesting no impact of *mlaA* deletion on OM permeability. Upon exposure to increasing concentrations of 3’,6-dinonyl neamine (0–10 µM), an increase in OM permeability was seen for both strains with a significant effect observed at 6 µM. At 8 µM and 10 µM, the effect was significantly higher for WT compared with ∆*mlaA* strain (26% and 22%, respectively) (Fig. 6A). The decrease in LPS and the larger Young’s modulus we observed for *P. aeruginosa* ∆*mlaA* could explain the lower susceptibility of the OM to be permeabilized by 3’,6-dinonyl neamine.

The IM permeability (Fig. 6B) was studied by propidium iodide, which fluoresces when it binds DNA. In the absence of an exogenous compound, no difference was observed for WT and *P. aeruginosa* ∆*mlaA*. 3’,6-dinonyl neamine induced a dose-dependent (0–10 µM) increase in IM permeability in both strains. At 6 µM and 8 µM, a significant effect was observed for ∆*mlaA* and WT strains, respectively. In contrast with data for OM, no difference in IM permeabilization was observed between WT and ∆*mlaA,* although the effect seemed slightly higher for *P. aeruginosa* ∆*mlaA* (Fig. 6B).

### Deletion of *mlaA* induced a decrease (2-fold to 8-fold) in MIC of fluoroquinolones

To tentatively link membrane permeability to the antimicrobial activity of antibiotics, we determined the MICs in *P. aeruginosa* WT and ∆*mlaA* for several antibiotics (abbreviations defined in Table 2). These included glycopeptides (VAN), lipoglycopeptides (TEL, DAP), oxazolidinone (LZD), cephalosporins (FEP, CAZ), penicillin (TMO), fluoroquinolones (CIP, LVX, MOX), polymyxin (COL), aminoglycoside (AMK, GEN, TOB, NEO), an amphiphilic aminoglycoside (3’,6-dinonyl neamine), carbapenems (IMI, MEM), rifamycin (RIF), macrolide (ERY), and aminocoumarin (NOV) (Table 2). Interestingly, fluoroquinolones have a MIC value lower (2-fold to 8-fold) against *P. aeruginosa* ∆*mlaA* compared with WT, a decrease that was reversed in complemented strain. In contrast, no major change was observed for cephalosporins, polymyxin E, aminoglycoside (conventional or amphiphilic), and carbapenem. Also, antibiotics that were inefficient against *P. aeruginosa* WT, such as penicillin, glycopeptide, lipoglycopeptides, oxazolidinone, rifamycin, macrolide, and aminocoumarin, showed no decrease in MIC values in ∆*mlaA* strain, suggesting that the sensitivity to antimicrobial compounds cannot be attributed to a global impairment of the OM/IM permeability. Changes in physicochemical parameters (i.e., hydrophobicity) could optimize the diffusion of some antibiotics, like fluoroquinolones, resulting in appropriate intracellular drug concentrations for antibacterial activity. A change in membrane fluidity might also impact the function of fluoroquinolone-specific efflux pumps or porins.

**TABLE 2:** Antibiotic susceptibility: MIC of different antibiotics determined for *P. aeruginosa* WT and ∆*mlaA* (*N* = 3) , ND not determined

	Antibiotics	Generic abbreviation	Target/Process	MICs (µg/mL)	
				Wild-type	∆mlaA
Aminoglycosides	Amikacin	AMK	Binding to the aminoacyl site of 16S ribosomal RNA within the 30S ribosomal subunit	8	8
	Gentamicin	GEN		4	4
	Tobramycin	TOB		1	2
	Neomycin	NEO		128	>128
	Neamine	NEA		>512	>512
Amphiphilic aminoglycoside derivative	3’,6-dinonyl neamine	3’,6-diNn	Binding to the LPS & GPL domains	4	4
Polymyxin	Colistin	COL	Binding to the LPS	4	2
Fluoroquinolones	Ciprofloxacin	CIP	Inhibiting DNA gyrase or topoisomerase IV	0.25	0.125
	Levofloxacin	LVX			
				1	0.25
	Moxifloxacin	MXF		2	0.5
Carbapenems	Imipenem	IPM	Binding to the penicillin-binding proteins	4	4
	Meropenem	MEM		0.5	1
Cephalosporins	Cefepime	FEP	Inhibiting penicillin-sensitive enzymes (transpeptidases, carboxypeptidases)	2	2
	Ceftazidime	CAZ		1	2
Penicillin	Temocillin	TMO	Inhibiting the transpeptidase that catalyzes the final step in cell wall biosynthesis	>64	>64
Glycopeptide	Vancomycin	VAN	Binding to the acyl-d-alanyl-d-alanine terminus of the growing peptidoglycan	>512	>512
Lipoglycopeptides	Telavancin	TEL	Inserting the lipophilic tail into the bacterial cell membrane causes rapid membrane depolarization and a potassium ion efflux	>512	>512
	Daptomycin	DAP		>512	>512
Rifamycin	Rifampicin	RIF	Inhibiting bacterial DNA-dependent RNA polymerase	16	16
Macrolide	Erythromycin	ERY	Binding to the 23S ribosomal RNA molecule in the 50S subunit of ribosomes	>64	>64
Aminocoumarin	Novobiocin	NVB	Inhibiting GyrB subunit of the bacterial DNA gyrase	>64	>64
Oxazolidinone	Linezolid	LZD	Binding a site on the bacterial 23S ribosomal RNA of the 50S subunit	0.5	1

### Deletion of *mlaA* increased *P. aeruginosa* membrane vesiculation. 3’,6-dinonyl neamine increased MVs number in WT and to a lesser extent in ∆*mlaA* strain

**Fig 7:**
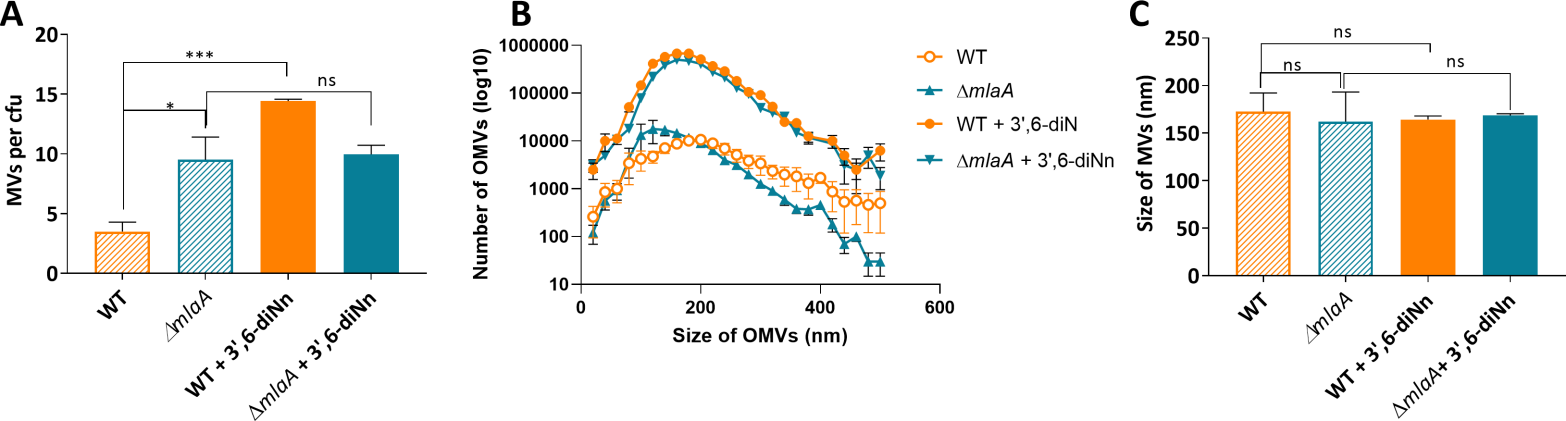
MVs quantification. MVs *P. aeruginosa* WT and ∆*mlaA* in the presence and absence of 3’,6-dinonyl neamine (1× MIC, 2 mg/L, 1 h) were analyzed using a nanoparticle tracking analyzer. (**A**) Number of MVs produced per colony forming unit (CFU). (**B**) MVs size distribution in nanometers (nm). Figure 6B was used to calculate the mean size of the membrane vesicles, which is shown in (**C**). Statistical analysis was performed by one-way ANOVA with Tukey’s multiple-comparison test. ****P < 0.0001, **P < 0.01, *P < 0.05; ^ns^*p* >0.05.

The differences in *P. aeruginosa* WT and ∆*mlaA* concerning their membrane lipid composition (Fig. 1 and 2) as well as differences in Young’s modulus (Fig. 3D) motivated us to investigate the formation of membrane-derived vesicles. The results showed that ∆*mlaA* produced more MVs (2.7-fold) compared with WT strain (Fig. 7A). This increase was largely reversed in complemented strain (Fig. S7B). The incubation of *P. aeruginosa* WT with 3’,6-dinonyl neamine markedly increased the MVs production with an increase of 4.1-fold (Fig. 7A) with no effect on ∆*mlaA*.

Regarding the MVs size distribution (Fig. 7B), no difference in the average mean size was observed when comparing MVs generated from WT (172.1 ± 19.5 nm) and ∆*mlaA* (162.3 ± 30.9 nm) strains (Fig. 7C). The addition of 3’,6-dinonyl neamine to WT (164.4 ± 3.8 nm) and ∆*mlaA* (168.5 ± 1.7 nm) did not change mean size of MVs.

## DISCUSSION

The maintenance of OM lipid asymmetry is critical for resisting antibiotics and host stresses, as reported for numerous Gram-negative bacteria including *E. coli* (60), *H. influenzae* (61), *Burkholderia cepacia* complex structures (8), *A. baumannii* (62), and *P. aeruginosa* (10, 63). LPS levels and localization are involved in preserving the stiffness and permeability barrier of Gram-negative bacteria (64). As such, the Mla (maintenance of the OM lipid asymmetry) transport, composed of MlaA/VacJ-MlaCBDEF and facilitating the retrograde phospholipid transport from the outer back to the inner membrane (11), has been proposed as a target for drug development (14).

Here, we focused on MlaA, contributing to removing phospholipids from the outer leaflet of OM and transferring them to MlaC located in the periplasm. We explored the consequences of *mlaA* deletion on the mechanical (cell stiffness, turgor pressure), biophysical (surface charge, fluidity), and functional (MVs generation, antibiotic susceptibility, permeability) properties of the OM in relation to changes in GPLs and LPS. All experiments were conducted in the absence and presence of one amphiphilic aminoglycoside, 3’,6-dinonyl neamine, known to interact with *P. aeruginosa* OM, and especially with LPS and GPLs (29–31, 65).

First, the work shows the changes in membrane lipid composition induced by *P. aeruginosa* to defend against the deletion of *mlaA.* Total GPLs increased with a prominent effect on PE. In parallel, bacterial LPS levels decreased, and changes in lipid A structures (arabinosylation, palmitoylation) were observed. These changes were associated with increased cell stiffness without alteration of turgor pressure and height profile. Remodeling of OM induced by *mlaA* deletion induced an increase in MVs generation without any significant effect on either IM fluidity or the IM/OM membrane permeability.

In the presence of 3',6-dinonyl neamine, *P. aeruginosa* WT also showed an increase in GPLs and a decrease in LPS with lipid A modifications (arabinosylation and palmitoylation), but differences of *P. aeruginosa* WT and ∆*mlaA* were observed with no significant effect on GPLs, a huge increase in LPS (associated with a decrease in lipid A hydroxylation and palmitoylation), decrease in cell stiffness and turgor pressure, and no effect on IM fluidity observed in *∆mlaA* strain. This might result in a slight and non-significant increase in MVs generation and less permeabilization of the OM in ∆*mlaA* as observed in WT strain. These differences of *P. aeruginosa* WT and ∆*mlaA* in response to 3',6-dinonyl neamine might result not only from the extent of changes in GPLs and LPS contents but also from changes in lipid A structures, which may result in differences in drug lipid insertion.

At a glance, the study first highlighted how *P. aeruginosa* copes with *mlaA* deletion and modifies its membrane lipid composition. The data confirm a link between maintaining OM lipid asymmetry and regulating the level of LPS molecules at the surface of Gram-negative bacteria (11, 17, 60). Intriguingly, there is a decrease in LPS contents in ∆*mlaA* compared with *P. aeruginosa* WT. The first hypothesis was the impact of *mlaA* deletion on LPS synthesis. We excluded a decrease in the stability of LpxC, one of the nine critical enzymes involved in lipid A synthesis, related to a partial or complete loss of YejM activity (64, 66, 67), since YejM was overexpressed in *P. aeruginosa* ∆*mlaA* (Fig. S4A), in agreement with Guest and co-authors (64). Whether a decrease in lipid A synthesis could be afforded by a reduced LpxK activity induced by changes in the ratio of unsaturated and saturated fatty acids (68) or a competition for a common substrate for GPLs and LPS synthesis, that is, β-hydroxymyristoyl–ACP (69, 70), is unknown. We also ruled out a restriction of LPS transport to OM related to a decrease in CL synthesis (69, 71, 72) since we did not observe any major change in cardiolipin content in *P. aeruginosa* ∆*mlaA* compared with WT (Fig. S3A). Finally, an increased release of LPS from the MVs in *P. aeruginosa* ∆*mlaA* compared with WT must be also excluded (Fig. S8B).

Interestingly, LPS and GPLs varied in the opposite way. Since MVs are enriched in GPLs and not in LPS (Fig. S8A), they are unlikely involved in maintaining the ratio between LPS and GPLs. One other explanation could be changes in the directionality of phospholipid transport (from IM to OM-anterograde transport and from OM to IM-retrograde transport), even anterograde transport described for *A. baumannii* (62, 73) and *E. coli* (33), is largely disputed. For *P. aeruginosa,* to our knowledge, nothing has yet been published.

Beyond the effect induced by *mlaA* deletion on LPS levels and the ratio between LPS and GPLs, an effect on lysophospholipids was also observed with a higher LPG content compared with LPE. This could result (i) from a lack of activity of PldA and PagP toward PE, (ii) a higher stability of OM when PE flipped due to its zwitterionic character and small headgroup size (74), and (iii) a higher release of LPE in MVs derived from ∆*mlaA* strain. In *P. aeruginosa* ATCC27853 WT and ∆*mlaA*, PCR products of *pagP* and *pldA* were present (Fig. 2B; Fig. S4C). However, a significant increase in mRNA expression was observed only in the case of *pagP* (2.7-fold) (Fig. 2B; Fig. S4C).

Second, the consequences of *mlaA* deletion on the mechanical and biophysical properties of *P. aeruginosa* membranes were evidenced. *P. aeruginosa* ∆*mlaA* showed an increase in cell surface stiffness likely linked with changes in lipid composition, including (i) longer acyl chains increasing the interactions between neighboring LPS molecules (3) and (ii) increase of phospholipids containing saturated acyl chains. However, the relationship between OM lipid composition and OM stiffness might be more complex, implying the presence of peptidoglycan and/or the link between peptidoglycan and OM (75, 76). Drugs interacting with LPS and/or GPLs like 3’,6-dinonyl neamine also played a critical role in the mechanoproperties of bacterial membranes by decreasing cell stiffness and turgor pressure independently of the *mlaA* deletion or not. This could result from competition with Ca^2+^ and interaction with negatively-charged LPS, which could resolve the destabilization of the OM. No change was observed for the IM fluidity by comparing *P. aeruginosa* ∆*mlaA* with WT, but a decrease of the surface charge was noticed, in agreement with lipid A modifications, including arabinosylation.

Third, focusing on the functional impact of *mlaA* deletion, a huge increase in the generation of MVs was observed in *P. aeruginosa* ∆*mlaA*. This is in agreement with literature (77–80) and complements data reported on *Haemophilus influenzae, Vibrio cholera* (80)*, Neisseria gonorrhoae* (77), *Campylobacter jejuni* (79), and *E. coli mlaA* knockouts (81). Increased vesiculation might provide a transient advantage during the adaptation process. MlaA, by regulating vesiculation, could allow bacteria to spare the consumption of energy requested by the continuous liberation of compounds of the OM and periplasm (80). The mechanism responsible for hypervesiculation in the ∆*mlaA* strain is likely complex. Curvature inducing lipid molecules (negative and positive) at particular sites might favor MVs release from bacteria (34). Since we observed an increase in PE (Fig. S3A), which displays positive and negative intrinsic molecular curvatures, respectively (82–84), we could imagine the formation of highly curved structures such as membrane vesicles. However, other hypotheses might be raised like (i) weaker interactions between LPS molecules resulting from the insertion of GPLs into the outer leaflet of OM, favoring thus OM bulging and formation of vesicles (85), (ii) changes in thermotropic behavior and modifications of fluidity resulting from changes in unsaturated/saturated lipid acyl chain ratio (86, 87) or changes in the polar head may facilitate membrane vesiculation. The PE, PG, and CL ratio might be critical in this respect as the phase transition temperature (Tm) of PE (DPPE, 41°C) is lower than that of PG (DPPG, 63°C). Also, CL is a lipid-containing four associated fatty acyl chains characterized by a high degree of unsaturation (88), which can form very rigid, gel-like membrane domains in the otherwise more fluid membrane promoting the initiation of vesicle formation (89). In addition, lipid A modifications resulting from environmental changes and PhoPQ-PmrAB activation (Fig. 2B), potentially interleaflet coupling with modified acylated lipid A and the pre-existing covalent linkages to peptidoglycans, could induce an increase of MVs production as reported for *Salmonella* (90). A last hypothesis is the accumulation of the PQS signaling molecule (2-heptyl-3-hydroxy-4-quinolone) in the outer leaflet of the OM. An increase in PQS was reported in *P. aeruginosa* ∆*mlaA* compared with the WT (5), and this might promote OM curvature and vesiculation (91, 92). Besides these effects, MVs generation could also be influenced by changes in the covalent OM-peptidoglycan crosslinks resulting from integral proteins (i.e., Lpp) (93).

In the presence of 3',6-dinonyl neamine, a huge enhancement in the generation of MVs was noticed, especially for *P. aeruginosa* WT. Modulating the LPS biosynthesis, changes in lipid A structure might directly facilitate the formation of MVs or favor the insertion of the drug, promoting MVs generation (38, 94). The reduced effect on ∆*mlaA* strains could be related to the lower content in LPS in comparison to WT or changes in lipid A modifications. This could be responsible for changes in the insertion of 3’,6-dinonyl neamine into the outer leaflet of the OM with an increase in the surface area of the outer leaflet, leading to enhanced interleaflet tension that is relieved through induction of membrane curvature and subsequent MVs release. This agrees with mechanisms behind the MVs biogenesis that were previously described (82, 91).

In the end, the question of the potential impact on antibiotic susceptibility was raised. The reduced LPS levels in the Δ*mlaA* strain were not sufficient to modify sensitivity to antibiotics like colistin or amphiphilic aminoglycoside, in agreement with the absence of OM permeabilization induced by these compounds at their MIC values. Two hypotheses could be raised (i) the decreased LPS may contribute to raise bacterial surface hydrophobicity, therefore, jeopardizing the outcome of *P. aeruginosa* interaction with hydrophobic and lipophilic molecules, and (ii) LPS modifications such as the lipid A palmitoylation and amino-arabinosylation or aminoacylation of phospholipids as reported for *P. aeruginosa* (95, 96) may contribute to antimicrobial peptide resistance. These effects on LPS might explain why OM is less prone to be permeabilized in *P. aeruginosa* ∆*mlaA* compared with WT strain impairing the activity of antibiotics like colistin for which the OM permeabilization is required to reach IM and induce cell lysis (97). More generally, if sensitivity to antimicrobial compounds is unlikely to be due to a global impairment of OM/IM permeability, changes in physicochemical parameters (i.e., hydrophobicity) could optimize the diffusion of some antibiotics such as fluoroquinolones. This could lead to appropriate intracellular drug concentrations for antibacterial activity. A change in membrane fluidity could also affect the function of fluoroquinolone-specific efflux pumps or porins.

Moving to the potential interest of MlaA in clinics, since this study does not consider the complexity of biological, clinically relevant conditions (98–100), it is difficult to extrapolate our conclusions to the *in vivo* situation directly. Other *Pseudomonas* strains (PAO1, PA14) or clinical strains (from patients suffering from cystic fibrosis) should be selected. Especially, it would have been interesting to compare the *P. aeruginosa* clinical isolates from the different sites of infection and geographical areas to understand the role of MlaA in bacterial physiology and pathology in a healthcare setting. Interestingly, during lower respiratory tract infection in non-typeable *H. influenzae* (NTHi) modulation of outer leaflet of OM through increased expression of *mlaA* (*vacJ*) genes could minimize recognition by bactericidal anti-oligosaccharide antibodies (61).

In conclusion, this work highlights some aspects of the membrane dynamics of *P. aeruginosa:* (i) the cross-talk between LPS and glycerophospholipids mislocated at the outer leaflet of the OM of *P. aeruginosa* (34) and (ii) the role of the individual glycerophospholipids and lipid A structures for changes in mechanical properties of OM, including cell stiffness, turgor pressure, and IM fluidity. The impact of the changes in membrane lipid composition on biophysical and functional membrane properties, including vesiculation and permeability, was also assessed. Although it is risky to define mechanical hypotheses linking lipid composition and membrane biophysical properties, especially since the system maintaining OM asymmetry is complex and involves several proteins probably acting in a coordinated manner, a better understanding of membrane biophysics as the maintenance of OM lipid asymmetry in link with lipid membrane composition is a pre-requisite for opening new research avenues in the design of innovative antibacterial drugs.

## MATERIALS AND METHODS

3’,6-dinonyl neamine (3’,6-diNn) was synthesized by Decout and colleagues (101). *P. aeruginosa* strain ATCC27853 (WT) was obtained from the Pasteur Institute (Brussels, Belgium; Prof. R. Vanhoof). Muller-Hinton broth cation-adjusted (MHB-Ca) and Luria-Bertani broth (LB) were purchased from Roth^TM^. LPE-17:1, LPG-17:1, and CL-14:0, used as internal standards, were purchased from Avanti Polar Lipids. FAME standards were ordered from Larodan (Solna, Sweden). CH_3_CN, CHCl_3_, CH_3_OH, and hexane from Fisher Scientific (Leicestershire, UK) were of high-performance liquid chromatography (HPLC) grade and were used without further purification. All other reagents and chemicals used were of the highest grade of purity commercially available. Milli-Q H_2_O was filtered through a 0.22 µm filter and obtained using a Milli-Q Millipore system (Milli-Q plus 185).

### Bacterial strains and culture conditions

Strains were grown on Tryptic Soy Agar (TSA), Luria-Bertani (LB) agar plates, or in LB media. *P. aeruginosa* ∆*mlaA* (PA_2800) mutant was created using two-step allelic exchange (102–104) as described earlier (5). Complementation was carried out to ensure that the deletion of the *mlaA* gene did not have a polar effect on adjacent genes or mutations elsewhere in the genome during the construction process. Wild-type *mlaA* under the control of its native promoter (P*mlaA*) was cloned into the mini-Tn7 vector, which was then used to integrate a single copy of P*mlaA-mlaA* at the *att*Tn7 site into the chromosome of *P. aeruginosa* ATCC27853 Δ*mlaA* by transposition as previously described in Choi and Schweizer (105).

*P. aeruginosa* ATCC27853 and *P. aeruginosa* ∆*mlaA* strains grew until the mid-exponential phase. For some experiments, strains were then incubated with 3’,6-dinonyl neamine at its MIC (2 µg/mL) against *P. aeruginosa* for 1 h.

Some of the phenotypes with complemented strains *(*∆*mlaA att7::mlaA*) were characterized and found to be similar to those from WT for growth rate, PQS levels, motility including swarming, swimming and twitching (5), or MVs generation and MICs (this study).

### Quantitative reverse transcription-polymerase chain reaction (RT-qPCR)

RT-qPCR was done to quantify the mRNA transcripts from the WT, the ∆*mlaA, and the ∆mla att7::mlaA*. Total RNA was isolated using InviTrap Spin Universal RNA Minit Kit (INVITEK). DNA contamination was removed by TURBO DNA-*free*^TM^ kit from Invitrogen, and RNA was quantified using nanodrop (OD_260_). One microgram of total RNA was heated at 65°C for 15 min and then cooled down at 0°C, mixed with buffer, MgCl_2_, random hexamers, dNTP mix, RNase inhibitor, and reverse transcriptase to synthesize complementary DNA (cDNA) using first-strand cDNA synthesis Kit (ref 11483188001, Roche). Reverse transcription was performed on iCycleriQ (BIO-RAD ) with thermal cycler programming as follows: 25°C for 10 min; 42°C for 60 min; 99°C for 5 min, and 4°C for 5 min. Real-time PCR was performed with SYBR green (SsoAdvanced Universal SYBR Green Supermix, BIO- RAD) and using the primers specified in Table S1 using a CF96 Real-Time System (C1000 Touch Thermal cycler, BIO-RAD).

### Lipid analysis

The lipid content of the different samples was analyzed by LC-MS, as previously reported (56). Briefly, lipids from the membranes were analyzed after liquid/liquid extraction (CH_2_Cl_2_-CH_3_OH-H_2_O, 4:2:1, vol/vol/vol) under an acidic condition in the presence of internal standards (LPE-17:1, LPG-17:1, and CL-14:0). Phospholipid analysis was performed using a Xevo TQ-S (Waters, USA), and cardiolipin analysis was carried out on an LTQ-orbitrap (from Thermo Fisher Scientific).

For the phospholipids, an HSS LC-18 column 100 × 2.1 mm, 1.8 µm (Waters, USA) was used at a temperature of 40°C. The mobile phase consisted of a gradient between A: CH_3_OH-CH_3_CN (9:1, vol/vol) 75% H_2_O 25%; B: CH_3_OH-CH_3_CN (9:1, vol/vol) and C: CH_3_(CH_2_)_2_-2-OH, all containing ammonium acetate (5 mM). An ESI probe, operated in negative mode, was used for sample ionization. The mass spectrometer parameters were as follows: capillary voltage: 2.9kV; cone voltage: 70V; desolvation temperature: 400°C; desolvation gas flow: 1,000 L/h; cone gas flow: 150 L/h; and nebulizer: six bars.

For the analysis of cardiolipins, a Nucleosil C8 column 150 × 4 mm, 5 µm (Macherey-Nagel, GmbH & Co. KG) was used. The same mobile phase as used for the other phospholipids was selected. An ESI probe operated in negative mode was used for cardiolipin ionization. The mass spectrometer parameters were as follows: capillary voltage: −40V; capillary temperature: 275°C; tube lens voltage: −173V; sheath gas flow: 20AU; auxiliary gas flow: 10AU, and sweep gas flow: 8AU.

The relative quantification of the lipids was based on the ratio between the area under the curve (AUC) of the lipid structures and the AUC of the respective internal standard. The obtained data were normalized to the total lipid content determined via labeling the lipids with FM4-64.

### Lipopolysaccharides (LPS) extraction and quantification

To quantify LPS levels, LPS molecules were extracted from bacterial suspensions at mid-log growth phases and quantified by purpald assay, with data normalized to colony-forming units.

LPS was extracted from bacteria using an LPS extraction kit (iNtRON Biotechnology). Briefly, *P. aeruginosa* cells were harvested at 2978 g for 5 min. One milliliter of lysis buffer was added to the pellet and vortexed vigorously. Two hundred microliters of CHCl_3_ were added to the suspension, vortexed, and incubated at room temperature for 5 min. The suspension was centrifuged to pipette out the upper layer (400 µL). Eight hundred microliters of purification buffer were mixed, and LPS was precipitated by incubation at −20°C for 20 min. After centrifugation, the LPS pellet was washed with 70% ethanol and dissolved in 10 mM Tris-HCl. Samples were then lyophilized to get powder until further use.

LPS was quantified using purpald assay based on periodate oxidation of formaldehyde generated from 2-keto-3-deoxyoctonate (KDO) (106, 107). Briefly, 50 µL of LPS samples or standards (LPS from *P. aeruginosa* serotype 10, 0–1,200 µg/mL) was added to 50 µL of 32 mM NaIO_4_ in a 96-well plate and incubated for 25 min. Fifty microliters of 136 mM purpald reagent in 2 N NaOH were added to each well, followed by incubation for 20 min, and the addition of 50 µL of 64 mM NaIO_4._ After 20 min of incubation, absorbance was measured at 550 nm with a SpectraMax M3 microplate reader (Molecular Devices, Sunnyvale, CA, USA).

By using standard curve for quantification (determined for each independent assay), and for sake of comparison, the results are expressed in %. We considered as 100%, the value obtained for *P. aeruginosa* WT and untreated and the values obtained for ∆*mlaA* strain and for WT/∆*mlaA P. aeruginosa* treated with 3’,6-dinonyl neamine or colistin were compared to this value (100%).

### Lipid A sample preparation and analysis

From LPS extracts, lipid A samples were prepared by mild hydrolysis with 200 µL 1% acetic acid at 100°C for 2 h (108). The solution was left at room temperature to cool down, and 400  µL of CHCl_3_ and 200  µL of H_2_O were added. The solution was then centrifuged to obtain a bottom layer that was dried under N_2_ stream. Lipid A samples were resuspended in CHCl_3_:CH_3_OH (4:1, vol/vol) and analyzed by direct injection with an LTQ-orbitrap mass spectrometer (Thermo Fisher Scientific, Bleiswijk, The Netherlands) in negative mode with electrospray ionization. The samples were directly infused at a flow rate of 10 µL/min for 3 min. The MS conditions were: capillary temperature: 275°C, capillary voltage: –10 V, and tube lens: –125 V. Sheath gas, auxiliary gas, and sweep gas flow rates were set at 5 AU.

### Fatty acid extraction from lipid A and analysis

The lipid A extract was treated according to a procedure adapted from Myron Sasser (109). Briefly, after adding the internal standard C20:0 (Larodan, Solna, Sweden), the extract was saponified by adding NaOH, CH_3_OH, and water. The methylation of fatty acids was done by adding HCl, water, and CH_3_OH to the solution. FAMEs were extracted using a mixture of hexane and methyl tert-butyl ether (1/1; vol/vol). Before injection in gas chromatography, the extract (hexane, methyl tert-butyl ether) was purified by adding water and sodium hydroxide.

FAMEs were injected and separated by gas chromatography (GC) (Trace 2000; Thermo Finnigan, Milan, Italy) equipped with an autosampler (GC PAL, CTC Analytics, Zwingen, Switzerland) and a column Ultra 2 (25 m × 0.32 mm ID, film thickness 0.52 µm; Agilent Technologies, Santa Clara, CA, USA), continuously flowed with H_2_ as a carrier gas at a constant pressure of 60 kPa. The GC temperature program was the following: an initial temperature of 80°C, which increased to 170°C with a temperature slope of 25°C/min, then from 170°C to 300°C with a slope of 5°C/min. The temperature of 300°C was maintained for 2 min to clean the column. FAMEs were detected with flame ionization detector (Thermo Finnigan, Milan, Italy) kept at a constant temperature of 255°C and flowed by air (350 mL/min) and H_2_ (35 mL/min). An external standard composed of FAME C10:0, 3OH-C10:0, C12:0, 2OH-C12:0, 3OH-C12:0, C16:0, and C20:0 (Larodan, Solna, Sweden) was used to identify the unknown peaks with the retention time and quantify the peaks through the known concentrations. Chromatographs were processed by using ChromQuest 5.0 software (Thermo Finnigan, Milan, Italy). The results were normalized with the total LPS amount and expressed in µg.

### MVs isolation and characterization

*P. aeruginosa* WT and ∆*mlaA* strains were grown in one-fifth of the volume of Erlenmayer’s flasks until mid-logarithmic phase and treated with 3’, 6-dinonyl neamine (1× MIC, 2 µg/mL) for 1 h. The bacterial cells were pelleted by centrifugation at 2,978 g at 4°C (Eppendorf 5810 R centrifuge: A-4–62 rotor). The supernatant was filtered via a 0.45 µm polyvinyl difluorine (PVDF) filter (Whatman) and then ultracentrifuged at 150,000 g for 3 h at 4°C (Beckman^TM^; 80 Ti rotor). The supernatant was discarded, and the pellet was resuspended in 10 mM HEPES-0.85% NaCl buffer, pH7.2 (MVs buffer). The MVs were purified using gradient density of OptiPrep-iodixanol (Sigma-Aldrich) in MV buffer, as described previously (110). ZetaVIEW S/N 18–400 (x30 series, Inning am Ammersee, Germany) was used to determine the size distribution and the amount of the MVs.

### Outer membrane isolation

Outer membrane from *P. aeruginosa* WT and ∆*mlaA,* non-treated and treated with 3’,6-dinonyl neamine, was extracted using Mizuno and Kageyama’s protocol (111). Briefly, bacteria were grown in LB media at 37°C, until OD_620_: 0.8. Cell pellet (1.5 g) was suspended in 9 mL of 2 M sucrose, 10 mL of 0.1 M Tris-HCl (pH 7.8 at 25^°^C), 0.8 mL of 1% Na-EDTA ( ethylenediaminetetraacetic acid disodium salt) (pH 7.0), and 1.8 mL of 0.5% lysozyme and incubated at 37°C for 60 min. After 30 min of incubation, DNase (3 µg/mL) was added, and incubation was continued for a further 30 min. Samples were centrifuged at 15,000 g, for 15 min, at 37°C to remove the spheroplasts. The supernatant was ultracentrifuged at 100,000 g, 60 min, 4°C to recover the outer membranes. Total lipids and proteins were quantified with a fluorescent probe FM4-64 and BCA protein assay kit (Pierce, Thermoscientific), respectively.

The absence of IM as a contaminant was confirmed with NADH oxidase assay, an IM-specific enzyme. Upon time, OM extracted from *P. aeruginosa* WT strain showed no change in absorbance, whereas IM extracts from *P. aeruginosa* and *E. coli* decreased in absorbance reflecting NADH consumption (Fig. S1A). To further confirm the absence of IM, western blot against LepB, an IM protein, was performed using IM extracted from *E. coli* as a positive control. The WB did not show any band in OM of *P. aeruginosa,* whereas IM showed the presence of LepB (Fig. S1B).

### Atomic force microscopy (AFM)

#### Bacterial growth conditions

*P. aeruginosa* WT and Δ*mlaA* cells were cultured overnight in 10 mL of LB medium, at 37°C, under shaking. Stationary phase cultures were then diluted to an OD_600_ = 0.1 in the same culture medium and allowed to grow until the exponential phase (OD_600_ = 0.8) under shaking at 37°C. *P. aeruginosa* WT and Δ*mlaA* strains were treated with 1× MIC (2 µg/mL) 3’,6-dinonyl neamine for 1 h. The cells were then centrifuged (2,000 g, 5 min), washed and resuspended in 1× PBS. These washing and resuspension steps were repeated twice, and at the end, the cellular suspension was diluted 1:100 in PBS.

#### Sample preparation

AFM studies were conducted with bacteria immobilized on glass-bottom Petri dishes previously coated with Poly-L-Lysine (PLL). PLL coating was achieved by letting the dish in contact with PLL solution for 20 min at room temperature and after rinsing twice with PBS to remove non-attached PLL molecules. One milliliter of bacterial suspension was subsequently added to the PLL-coated dish and incubated for 20 min at room temperature. After that, at least three rinsing steps with PBS were performed, and the dish was filled with 2 mL of this buffer.

#### AFM imaging and mechanical studies

Both imaging and mechanical studies were carried out with MSCT-C cantilevers (Bruker), in PBS, at room temperature, using a JPK NanoWizard 4 NanoScience AFM. Before the experiments, the cantilevers were thoroughly rinsed with water and ethanol, dried with N_2_ flow, and further cleaned in a UV ozone chamber for 15 min. Freshly cleaned cantilevers were calibrated by the thermal noise method (112), and spring constant values were found to be always approximately 0.02 N m^−1^. Bacteria were first imaged in quantitative imaging (QI) mode at 128 × 128 pixels resolution, using an applied force of 0.5 nN, a constant approach/retract speed of 30 µm/s, and a ramp size of 500 nm. High-resolution (256 × 256 pixels) QI images were then recorded by imaging a 400 × 400 nm^2^ area on top of the bacteria, keeping the same imaging parameters. Mechanical measurements were conducted in force volume (FV) mode. For each cell, 256 force-distance curves (16 × 16 pixels) were recorded on 250 × 250 nm^2^ areas using an applied force of 1 nN, a constant approach/retract speed of 1 µm/s, and a ramp size of 1 µm. Data were analyzed with the JPK Data Processing software (version 6.1.172). The approach segment of the force-distance curves was fitted with the Hertz/Sneddon model over a distance of 20 nm, using a Poisson ratio of 0.5 and considering a conical tip of 15° half-cone angle (113). Although the Young’s Modulus (*E*) was extracted from the nonlinear region of the curves, the spring constant (*k*) was calculated through the slope of their linear region.

### Fluorescence-lifetime imaging microscopy (FLIM)

#### Sample preparation

*BODIPY-C10* was obtained from the Molecular Design and Synthesis lab at KU Leuven. The bacterial slide preparation protocol was adapted from previous studies with some modifications (56, 114). Briefly, the cells (10^7^ CFU/mL) were resuspended in the PBS containing BODIPY-C10 (0.5 µM) supplemented with 0.1% wt/vol of glucose. The cells were labeled by mixing and incubating at 37°C with shaking. Subsequently, 200 µL of the cell suspensions was immobilized on the 8-well Ibidi microscopy chamber, precoated with 0.01% poly-L-Lysine. BODIPY-C10 concentrates mostly in the inner membrane of *P. aeruginosa* (56).

#### FLIM imaging

The chambers containing the bacteria were mounted on a confocal & multiphoton microscope (LSM 980, ZEISS) equipped with a time-correlated single photon counting (TCSPC) system FLIM module (PicoQuant, Germany). BODIPY-C10 was excited with a coherent (Chameleon Discovery) pulsed laser (80 MHz) at 800 nm, and emission was recorded over a spectrum of 505–545 nm. The FLIM images were obtained at the resolution of 512 × 512 pixels.

The analysis of the FLIM images was performed with SymPhoTime 64 software, which allows fitting the fluorescence decay of the images’ pixels to mathematical decay models. An average lifetime is hence calculated for each pixel, which is color-coded in the FLIM images. Each decay curve and its corresponding image were acquired after collecting 1,000 photons.

### Membrane permeabilization by 3’, 6-dinonyl neamine

#### OM permeabilization

The OM permeabilization was assessed by the method used by Angus et al (115). Briefly, the overnight culture of *P. aeruginosa* was grown. 0.25 µg/mL of imipenem was added to the bacterial culture to induce a higher level of β-lactamases and incubated for an hour. Bacteria were centrifuged at 3,000 g for 7 min and then, resuspended in 1× PBS (pH 7.2) to get an OD_620_ 0.5. Thereafter, 50 µL of different concentrations (0–10 µM) of 3’, 6-dinonyl neamine was added to 100 µL bacterial suspension. Fifty microliters of nitrocefin (50 µg/mL) were added in the 96-well plates. Absorbance at 490 nm was monitored with a SpectraMax M3 microplate reader at 25°C (Molecular Devices, Sunnyvale, CA, USA). Ten micrometers of colistin was used as a positive control (100%).

#### IM permeability

The permeabilization of bacterial membranes was determined by using a membrane-impermeable probe, propidium iodide (PI), which fluoresces only when it binds to DNA (31). Briefly, an overnight culture of *P. aeruginosa* was grown and diluted to (OD_620_ 0.5) and added to PI (dissolved in water) and different concentrations of 3’,6-dinonyl neamine (0–10 µM). Suspension was incubated in dark for 15 min, and fluorescence intensity for excitation, 540 nm, and emission, 610 nm, was measured with a SpectraMax M3 microplate reader at 25°C (Molecular Devices, Sunnyvale, CA, USA). Ten micrometers of colistin were used as a positive control (100%).

### Antibiotic susceptibility assay

MICs of antibiotics were assessed by microdilution in cation-adjusted-Mueller Hinton Broth according to Clinical and Laboratory Standard Institute (2018).

### Zeta potential

Dynamic light scattering (DLS) technology was performed using a Zetasizer Nano SZ equipment from Malvern Instruments (Grovewood Road, UK) with patented NIBS (non-invasive back scatter) technology, and the recommended software was used for zeta potential determination.

## SUPPLEMENTAL MATERIAL

Figure S1 spectrum.01484-24-s0001.tiff (A) NADH oxidase activity upon time; (B)Western Blot; (C)PCR products of mlaA, mlaB, mlaC, mlaD, mlaE, and mlaF; (D)Relative mRNA expression in the ∆mlaA strain.

Figure S2 spectrum.01484-24-s0002.tiff Individual glycerophospholipids (PE, PG, CL) and lysophospholipids (LPE, LPG) species analysis.

Figure S3 spectrum.01484-24-s0003.tiff (A) Individual glycerophospholipids; (B) lysophosphoglycerides; (C) and saturated, monounsaturated, and polyunsaturated fatty acyl chains.

Figure S4 spectrum.01484-24-s0004.tiff mRNA expression.

Figure S5 spectrum.01484-24-s0005.tiff AFM height characterization of *P. aeruginosa* WT and ∆*mlaA* non-treated and treated.

Figure S6 spectrum.01484-24-s0006.tif Zeta potential of the bacterial envelope of *P. aeruginosa* WT and ∆*mlaA* strains.

Figure S7 spectrum.01484-24-s0007.tif Membrane vesicles (MVs) quantification.

Figure S8 spectrum.01484-24-s0008.tif Composition of Membrane vesicles (MVs) (n=3) with (A) GPLs and (B) LPS.

Tables S1 to S3 spectrum.01484-24-s0009.docx Table S1: Primers used. Table S2: Predicted lipid A acyl chains. Table S3: Relative intensity of lipid A structures.

Supplemental material spectrum.01484-24-s0010.docx Supplemental captions of figures and tables, and additional experimental details.

ASM does not own the copyrights to Supplemental Material that may be linked to, or accessed through, an article. The authors have granted ASM a non-exclusive, world-wide license to publish the Supplemental Material files. Please contact the corresponding author directly for reuse.
