# Supplementary figures and images for: Insight into the outer membrane asymmetry of P. aeruginosa and the role of MlaA in modulating the lipidic composition, mechanical, biophysical, and functional membrane properties of the cell envelope
Source: Microbiol Spectr. 2024 Oct 7;12(11):e01484-24. doi: 10.1128/spectrum.01484-24 (PMC11537012; doi:10.1128/spectrum.01484-24)

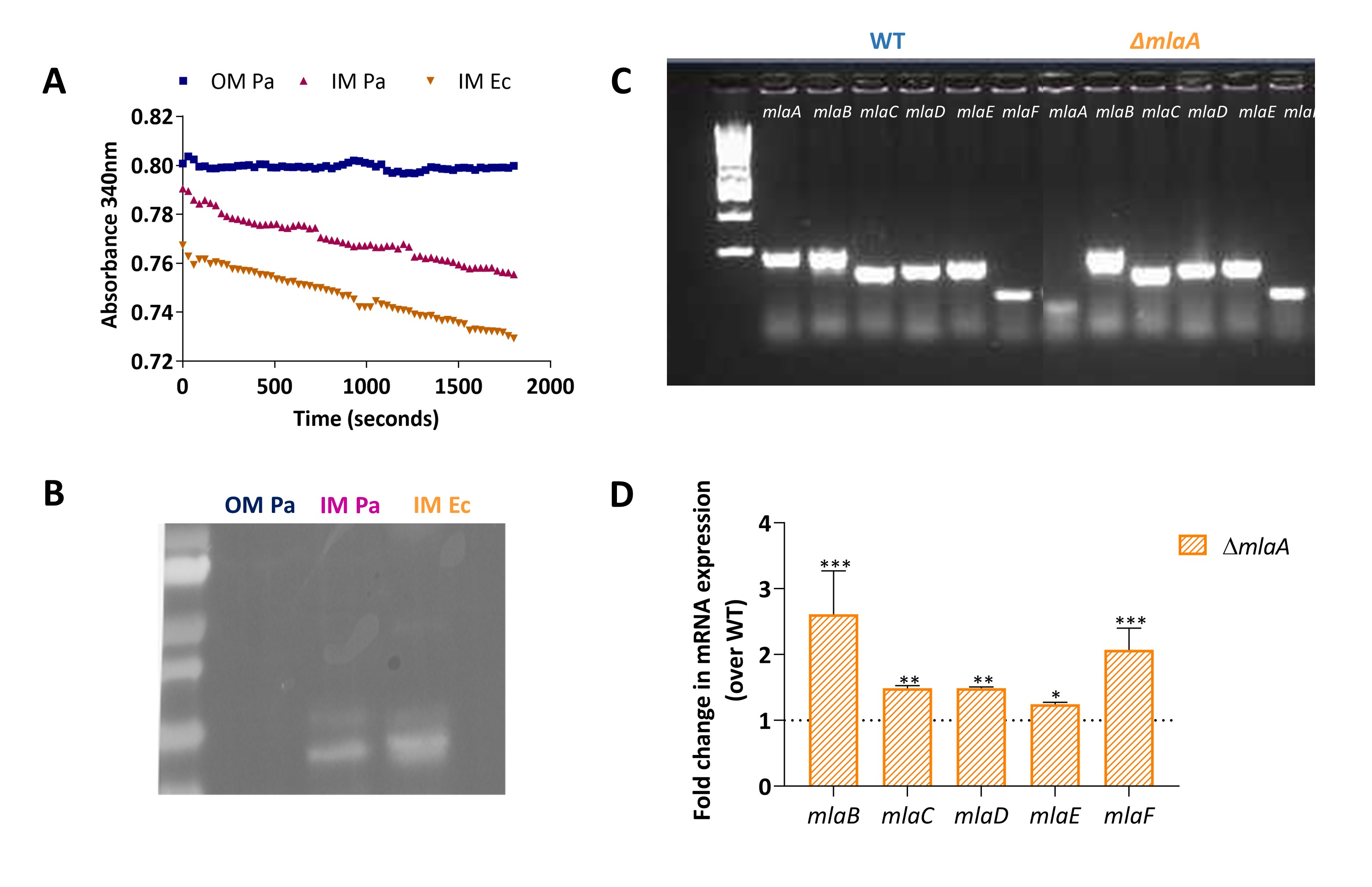

Supplement: Figure S1 — (A) NADH oxidase activity upon time; (B)Western Blot; (C)PCR products of mlaA, mlaB, mlaC, mlaD, mlaE, and mlaF; (D)Relative mRNA expression in the ∆mlaA strain. [file spectrum.01484-24-s0001.tiff]

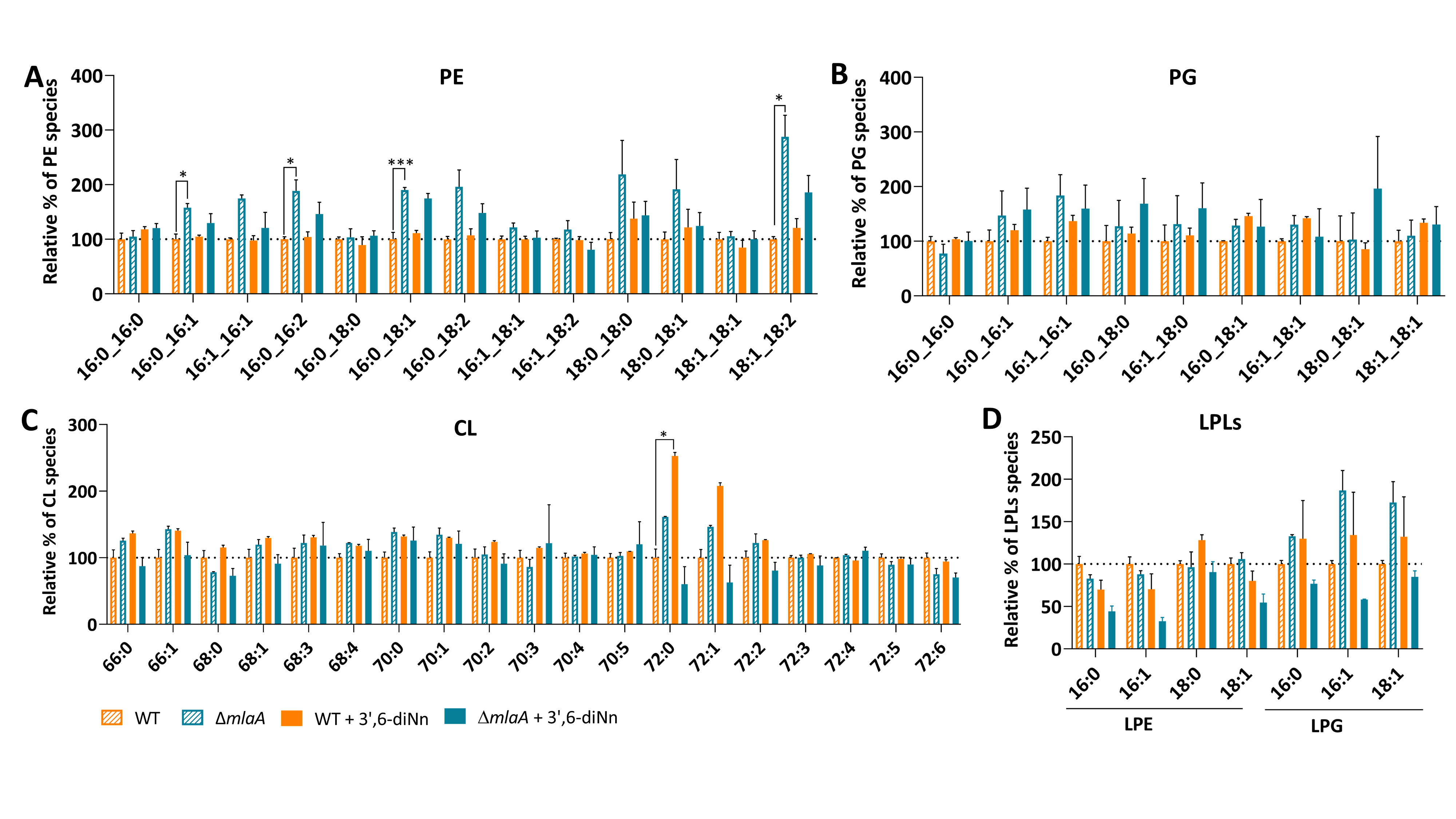

Supplement: Figure S2 — Individual glycerophospholipids (PE, PG, CL) and lysophospholipids (LPE, LPG) species analysis. [file spectrum.01484-24-s0002.tiff]

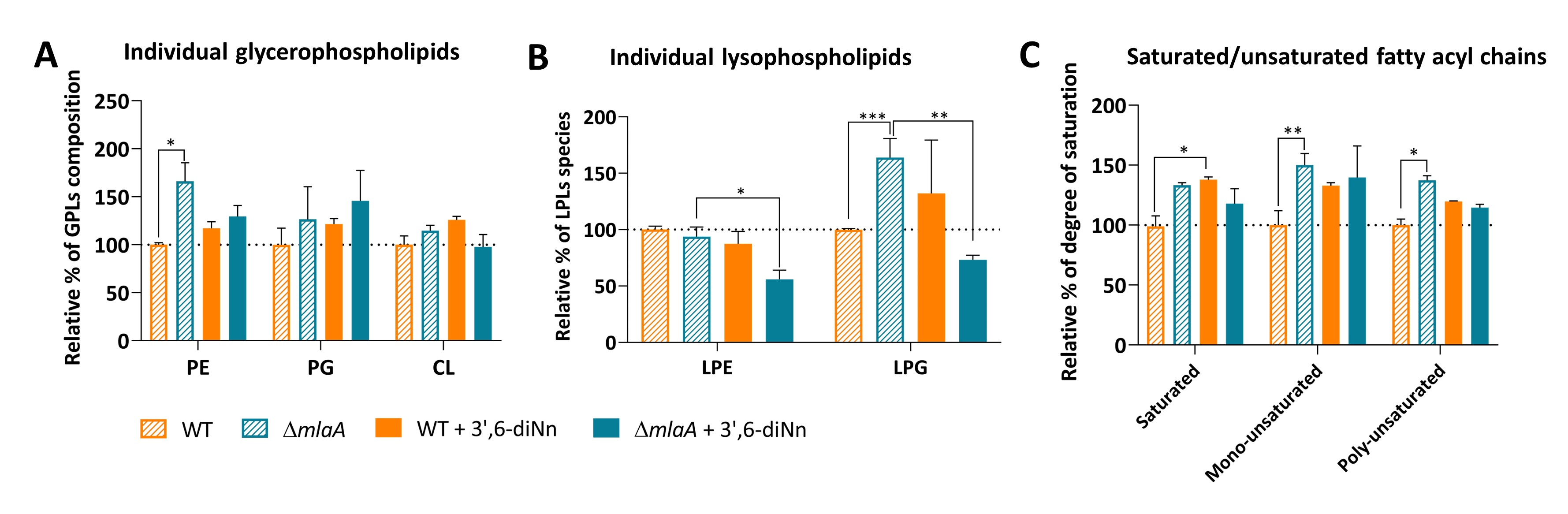

Supplement: Figure S3 — (A) Individual glycerophospholipids; (B) lysophosphoglycerides; (C) and saturated, monounsaturated, and polyunsaturated fatty acyl chains. [file spectrum.01484-24-s0003.tiff]

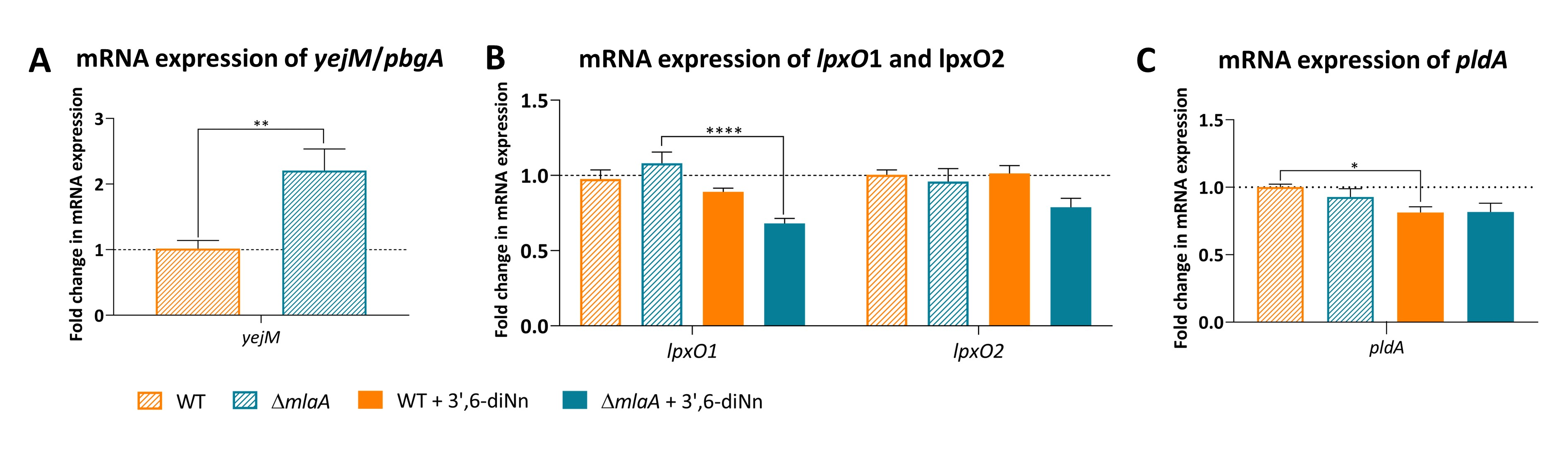

Supplement: Figure S4 — mRNA expression. [file spectrum.01484-24-s0004.tiff]

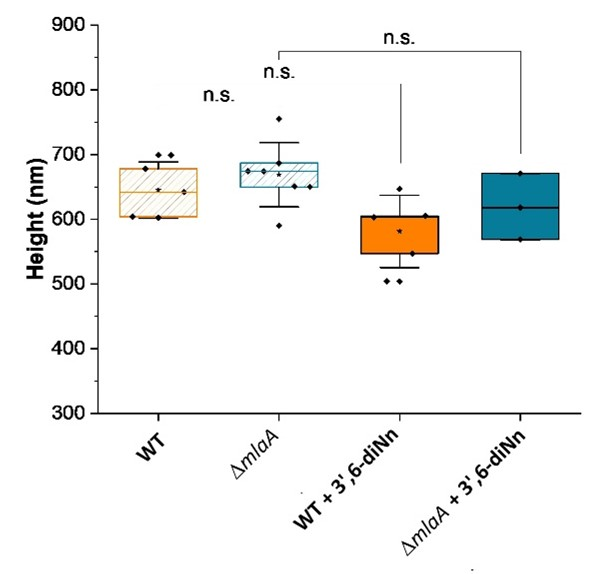

Supplement: Figure S5 — AFM height characterization of P. aeruginosa WT and ∆mlaA non-treated and treated. [file spectrum.01484-24-s0005.tiff]

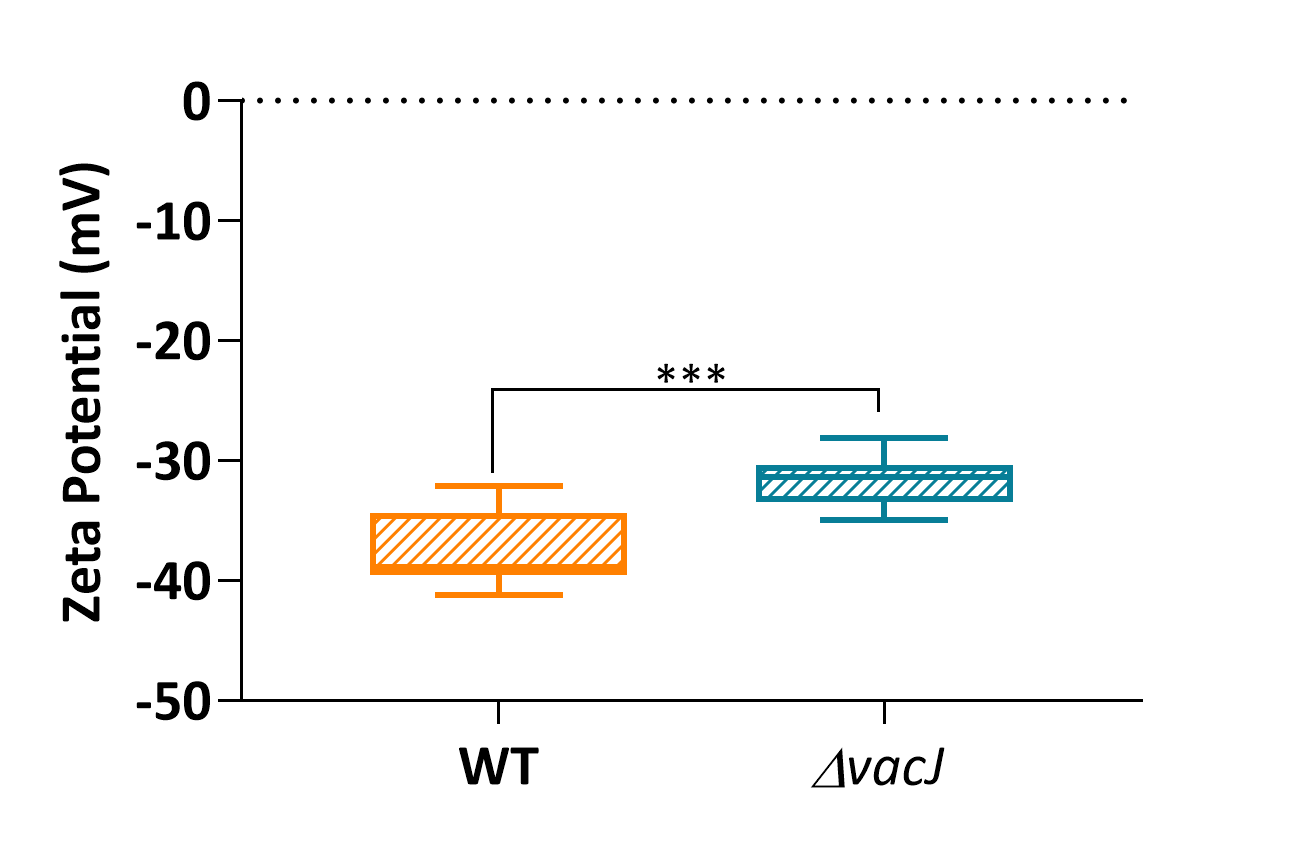

Supplement: Figure S6 — Zeta potential of the bacterial envelope of P. aeruginosa WT and ∆mlaA strains. [file spectrum.01484-24-s0006.tif]

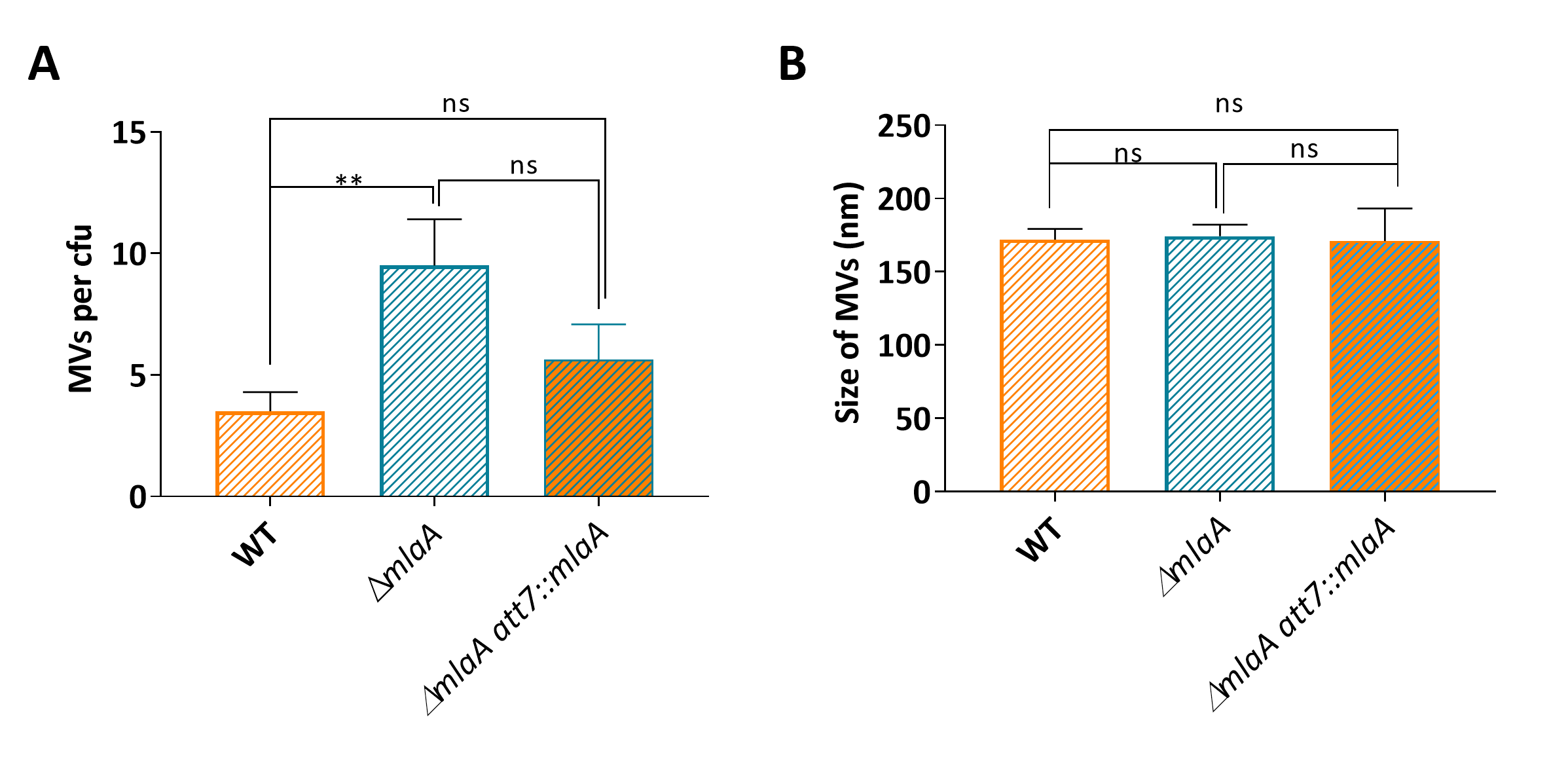

Supplement: Figure S7 — Membrane vesicles (MVs) quantification. [file spectrum.01484-24-s0007.tif]

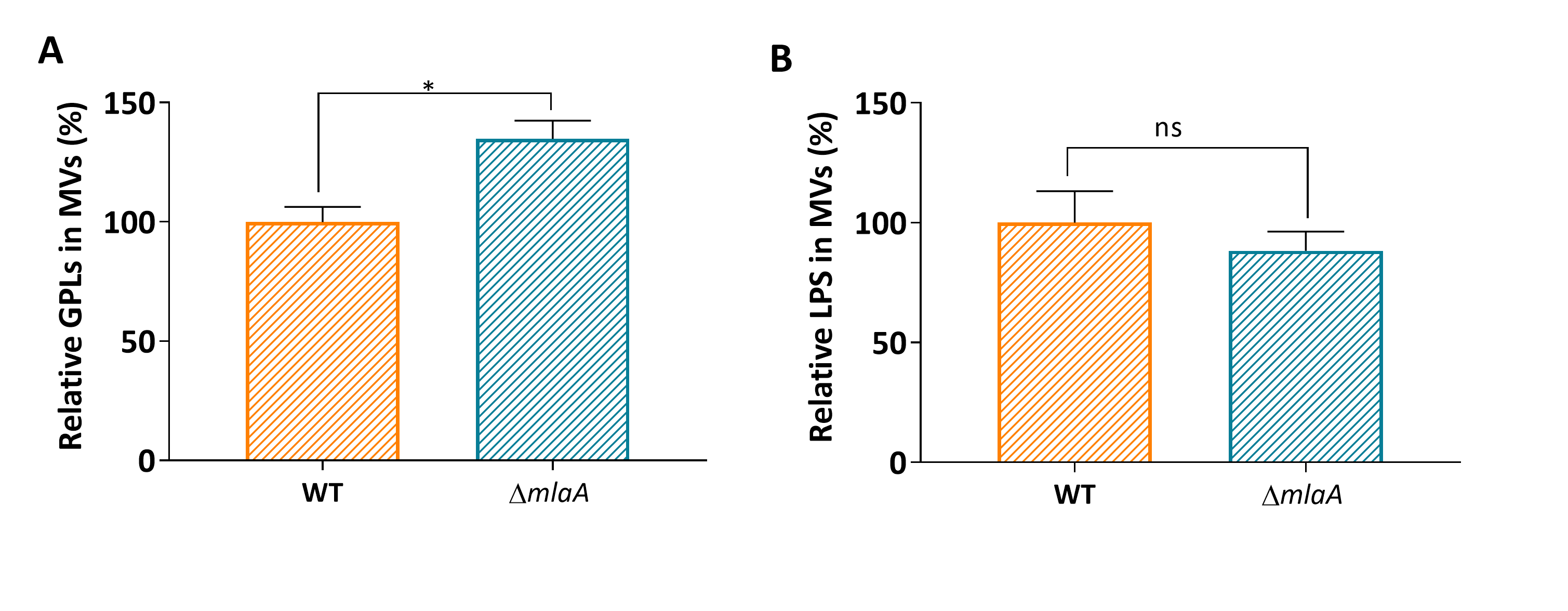

Supplement: Figure S8 — Composition of Membrane vesicles (MVs) (n=3) with (A) GPLs and (B) LPS. [file spectrum.01484-24-s0008.tif]
