# Supplementary material for: Insight into the outer membrane asymmetry of P. aeruginosa and the role of MlaA in modulating the lipidic composition, mechanical, biophysical, and functional membrane properties of the cell envelope
Source: Microbiol Spectr. 2024 Oct 7;12(11):e01484-24. doi: 10.1128/spectrum.01484-24 (PMC11537012; doi:10.1128/spectrum.01484-24)
Supplement: Tables S1 to S3 — Table S1: Primers used. Table S2: Predicted lipid A acyl chains. Table S3: Relative intensity of lipid A structures. [file spectrum.01484-24-s0009.docx]

| Table S1: Primers used in this study |
| --- |
|  |
| 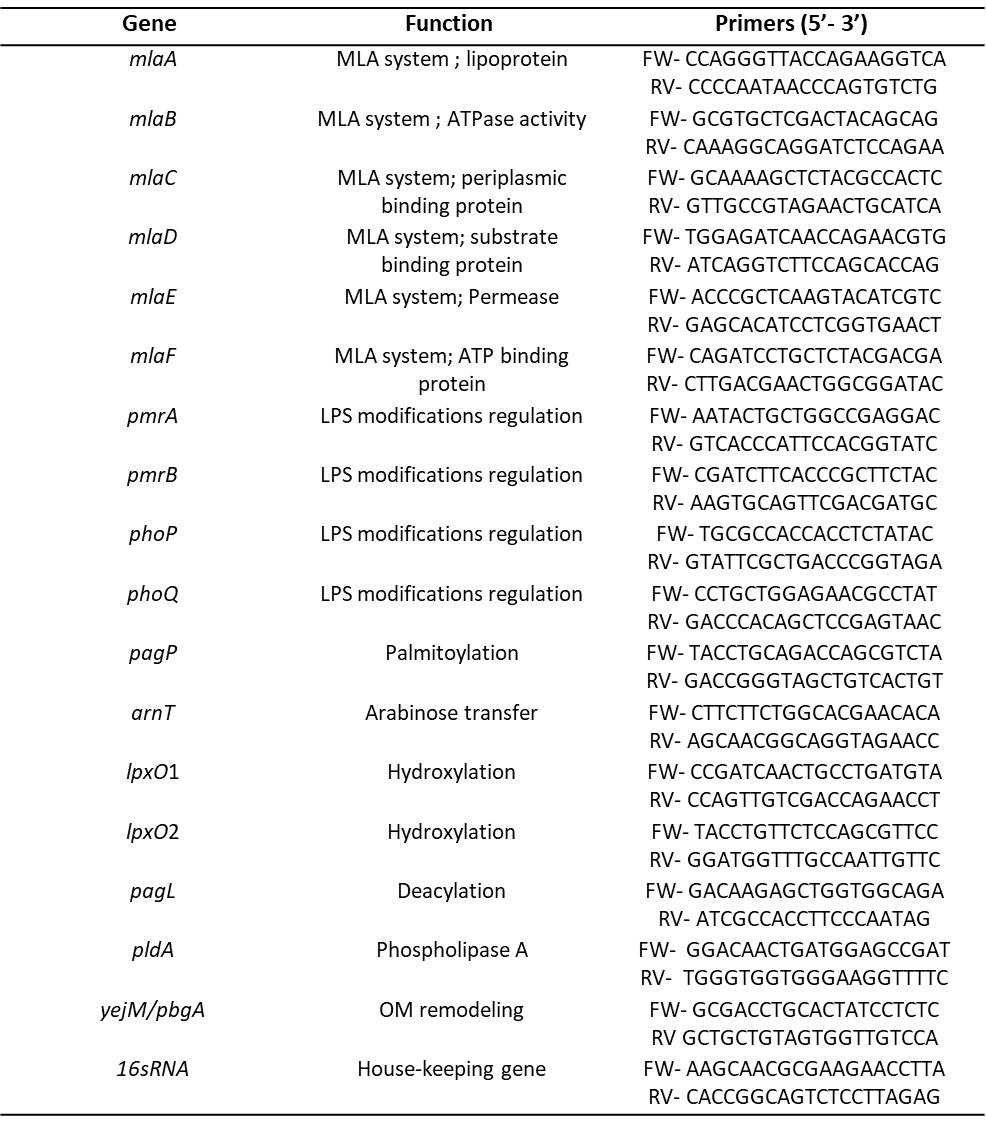 |

| Table S2: Predicted lipid A acyl chains where X = H or OH and positions can be interchanged | 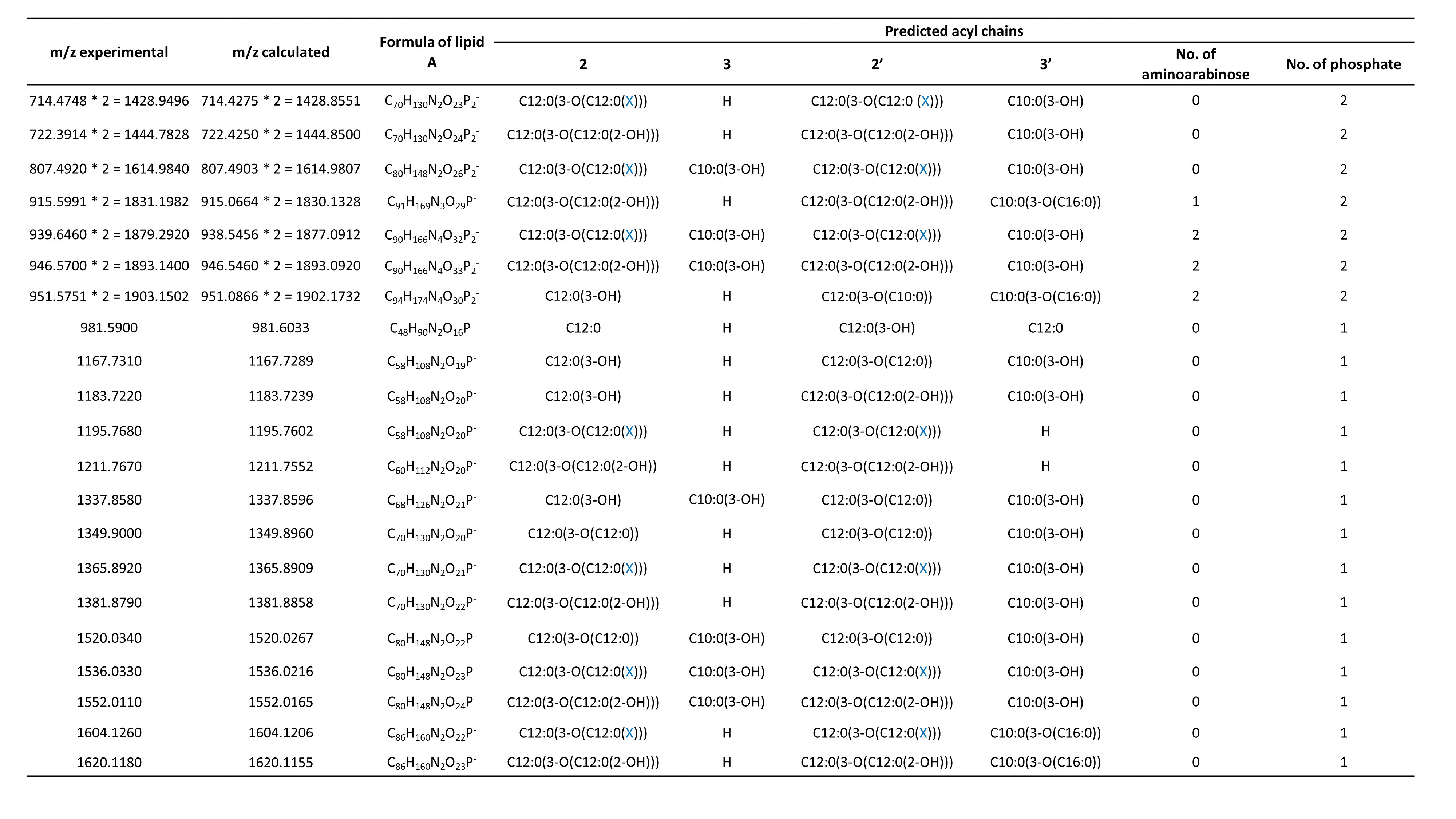 |
| --- | --- |

| Table S3: Relative intensity of lipid A structures. Statistics was performed by 2-way ANOVA with multiple-comparisons respectively, *****p* <0.0001, ****p* <0.001, ***p* <0.01, **p* <0.05; ^ns^*p* >0.05. a depicts significance between WT vs ∆*mlaA*, b depicts significance between WT vs WT + 3’,6-dinonyl neamine, c depicts significance between ∆*mlaA* vs ∆*mlaA* + 3’,6-dinonyl neamine, and ND is not detected. |
| --- |
| 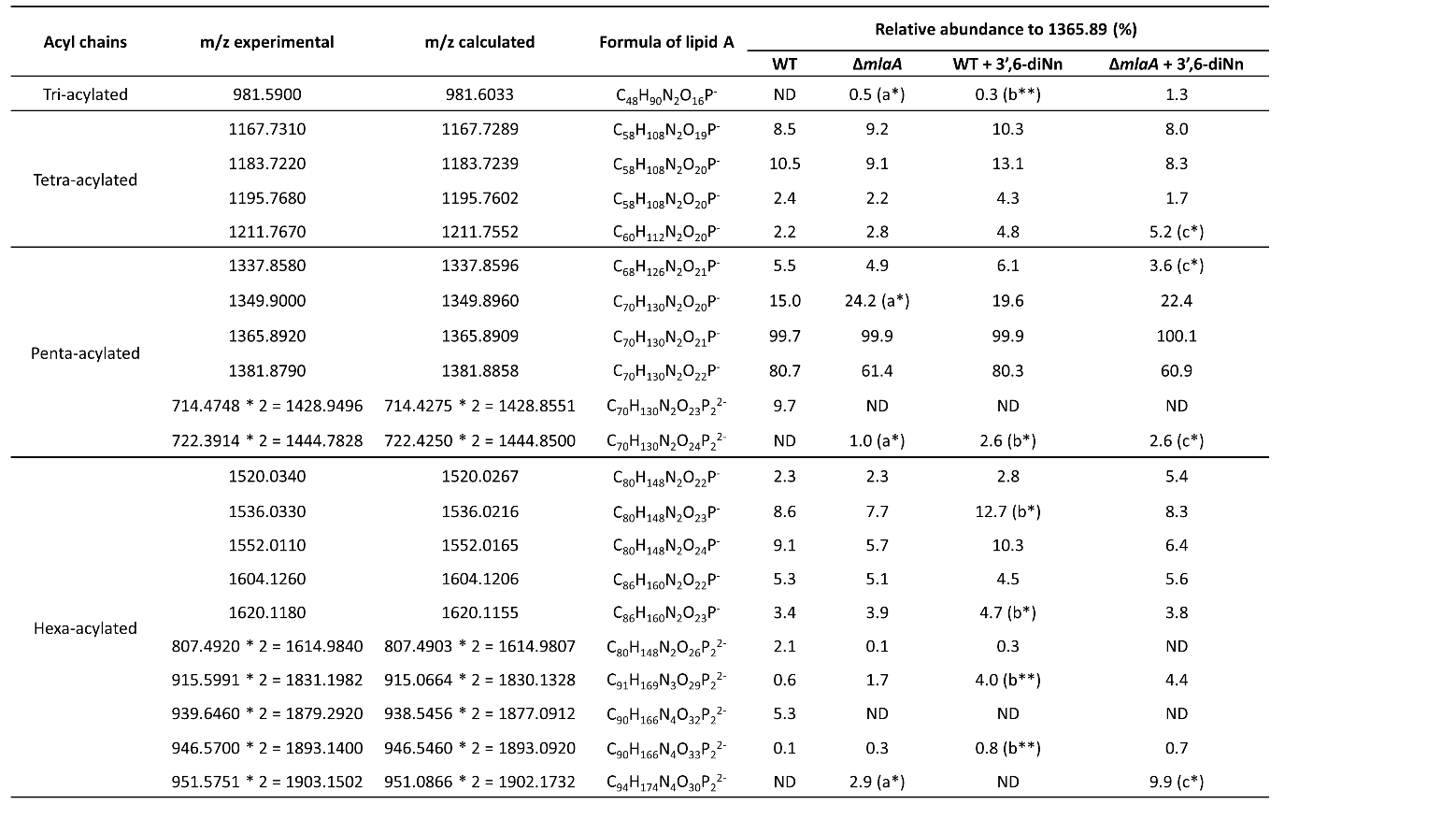 |
