# Supplementary material for: Insight into the outer membrane asymmetry of P. aeruginosa and the role of MlaA in modulating the lipidic composition, mechanical, biophysical, and functional membrane properties of the cell envelope
Source: Microbiol Spectr. 2024 Oct 7;12(11):e01484-24. doi: 10.1128/spectrum.01484-24 (PMC11537012; doi:10.1128/spectrum.01484-24)
Supplement: Supplemental material — Supplemental captions of figures and tables, and additional experimental details. [file spectrum.01484-24-s0010.docx]

**Table S1.** Primers used in this study

**Table S2.** Predicted lipid A acyl chains where X = H or OH and positions can be interchanged

**Table S3.** Relative intensity of lipid A structures. Statistics was performed by 2-way ANOVA with multiple-comparisons respectively, *****p* <0.0001, ****p* <0.001, ***p* <0.01, **p* <0.05; ^ns^*p* >0.05. a depicts significance between WT vs ∆*mlaA*, b depicts significance between WT vs WT + 3’,6-dinonyl neamine, c depicts significance between ∆*mlaA* vs ∆*mlaA* + 3’,6-dinonyl neamine, and ND is not detected.

**Fig. S1.** (A) NADH oxidase activity upon time as monitored by changes in OD_340nm_ for *P. aeruginosa* IM (pink triangles) and OM (blue squares) and from *E. coli* IM (yellow inverted triangles). (B) Western Blot against LepB, an IM protein in OM *P. aeruginosa*, IM *P. aeruginosa*, IM *E. coli* (control). (C) PCR products of *mlaA, mlaB, mlaC, mlaD, mlaE,* and *mlaF* in WT and ∆*mlaA* analyzed on 2% agarose gel by electrophoresis stained with gel red (N=3). (D) Relative mRNA expression in the ∆*mlaA* strain of the genes involved in the maintenance of OM lipid asymmetry over WT. Transcript levels of *mlaB, mlaC, mlaD, mlaE,* and *mlaF* were measured at exponential phase and normalized with a housekeeping gene; 16sRNA. (N=3, n=6) Statistical analysis was performed by multiple-t test *****p* <0.0001, ****p* <0.001, ***p* <0.01, **p* <0.05

**Fig.S2.** Individual glycerophospholipids (PE, PG, CL) and lysophospholipids (LPE, LPG) species analysis P. aeruginosa WT and ∆*mlaA* OM in the presence and absence of 3’,6-dinonyl neamine (1xMIC, 2µg/mL, 1h). Results are expressed in %, where WT is considered as 100%. Statistical analysis was performed by one-way ANOVA with multiple-comparisons *****p* <0.0001, ****p* <0.001, ***p* <0.01, **p* <0.05.

**Fig.S3.** (A) Individual glycerophospholipids, (B) lysophosphoglycerides, (C) and saturated, monounsaturated, and polyunsaturated fatty acyl chains in the OM of *P. aeruginosa* WT and ∆*mlaA* in the presence and absence of 3’,6-dinonyl neamine. PE, PG, CL, LPE, and LPG were analysed by LC-MS. Results are expressed in %, and *P. aeruginosa* WT is taken as 100%. Statistical analysis was performed by 2-way ANOVA with multiple-comparisons ****p* <0.001, ***p* <0.01, **p* <0.05.

**Fig.S4.** mRNA expression of (A) *yejM,* (B) *pldA* and (C) *lpxO1/O2* in *P. aeruginosa* WT and ∆*mlaA* in the presence and absence of 3’,6-dinonyl neamine. Statistics was performed by t-test, one-way ANOVA Tukey’s multiple-comparison test and 2-way ANOVA with multiple-comparisons respectively, *****p* <0.0001, ***p* <0.01, **p* <0.05.

**Fig. S5.** AFM height characterization of *P. aeruginosa* WT and ∆*mlaA* non-treated and treated with 3’,6-dinonyl neamine (1xMIC, 2µg/mL, 1h). Box plot showing the height values determined from AFM images for WT and Δ*mlaA* strains, before and after treatment with 3,6-dionyl neamine (1xMIC, 2µg/mL, 1hr). Stars indicate the mean values, lines the medians, boxes the 25-75 % quartiles and whiskers the standard deviation obtained from at least 3 independent cells over at least three independent experiments. ^ns^*p* > 0.01, determined by one-way ANOVA with Tukey’s multiple-comparison test ****p* <0.001, ***p* <0.01, **p* <0.05.

**Fig. S6.** Zeta potential of the bacterial envelope of *P. aeruginosa* WT and ∆*mlaA* strains (n= 9). Statistical analysis was performed by t- test, ****p* <0.001, ***p* <0.01, **p* <0.05.

**Fig.S7.** Membrane vesicles (MVs) quantification. (A). Number of MVs produced per colony forming unit (CFU) from *P. aeruginosa* WT, ∆*mlaA* and (∆*mlaA* att7⸬*mlaA*). (B). MVs size distribution *in P. aeruginosa* WT, ∆*mlaA* and (∆*mlaA* att7⸬*mlaA*) expressed in nanometers (nm). Statistical analysis was performed by one-way ANOVA with Tukey’s multiple-comparison test. *****p* <0.0001, ***p* <0.01, **p* <0.05; ^ns^ *p* >0.05.

**Fig.S8.** Composition of Membrane vesicles (MVs) (n=3) with (A) GPLs and (B) LPS. Statistical analysis was performed by t- test, ****p* <0.001, ***p* <0.01, **p* <0.05.

**Detailed changes in lipid A structures induced by *mlaA* deletion, in presence or not of 3’,6 dinonyl neamine**.

Based on the mass and fragmentation m/z of 1385.89 could contain one 3-OH C10:0, one C12:0, one 2-OH C12:0 and two 3-OH C12:0 as fatty acids. The positions of fatty acids and OH groups are hypothesized based on the structures given in (116). Along with it, two other major peaks, non-hydroxylated PLA1 (-16u) (*m/z* 1349.90, PLA2) and hydroxylated PLA1 (+ 16u) (*m/z* 1381.88 PLA3), penta-acylated forms of lipid A were also detected in all the samples. In addition, a smaller peak at *m/z* 1337.86 (PLA4) potentially corresponding to a penta-acylated lipid A was also identified that could correspond to one C12:0, two 3-OH C:10:0, and two 3-OH C12:0. Tetra-acylated lipid A *m/z* 1167.73 (possibly corresponding to PLA2-C12:0+H_2_O), 1183.72 (possibly corresponding to PLA3-C12:0 + H_2_O), 1195.77 (possibly corresponding to PLA1 - 3-OH C10:0 + H_2_O) were also found in samples with no statistical differences within the different conditions.

The absence of *mlaA* gave rise to 3 new peaks, a tri-acylated lipid A (m/z 981.59), a *bis*-phosphorylated penta-acylated lipid A (*m/z* 722.39, exact mass 1444.78), and a possibly PagP and ArnT derived hexa-acylated lipid A molecule with one additional palmitic ester and two additional L-Ara4N moieties (*m/z* 951.57, exact mass 1903.15). Increased signals of *mono*-phosphate penta-acylated lipid A (*m/z* 1349.90) compared to the WT strain was observed. Interestingly, a decrease, yet non-significant, in the signals of hydroxylated lipid A structures at *m/z* 1381, *m/z* 1552, and *m/z* 1536, unrelated to the expression of *lpxO1* and *lpxO2* (Fig S4B) was observed. In *P. aeruginosa* PAO1, decreased hydroxylation of lipid A is related to attenuated virulence (117) and potent induction of human cytokines (118). However, hydroxylation of lipid A in secondary acyl chains is not related to OM integrity in *P. aeruginosa* PAO1 (117). When comparing *P. aeruginosa* ∆*mlaA* to WT, *bis*-phosphorylated penta-acylated lipid A with *m/z* 714.48 (exact mass 1428.95) and *bis*-phosphorylated hexa-acylated lipid A with *m/z* 939.65 (exact mass 1879.29) disappeared.

After 3’,6-dinonyl neamine treatment, the intensity of the peaks associated to lipid A molecules in the WT and ∆*mlaA* varied. When 3’,6-dinonyl neamine was added to the WT strain, new peaks corresponding to the tri-acylated lipid A at *m/z* 981.59 and a *bis*-phosphorylated penta-acylated lipid A at *m/z* 722.39, exact mass 1444.78, emerged, while a *mono*-phosphorylated hexa-acylated lipid A (*m/z* 1536.03) and a *bis-*phosphorylated hexa-acylated lipid A with additional L-Ara4N moieties (m/z 946.57, exact mass 1893.14) significantly increased. In addition, an increase in the detection of *mono*-phosphorylated palmitoylated lipid A (*m/z* 1620.12, addition of C16:0 to *m/z* 1381.88 – PLA3 by PagP), and *bis-*phosphorylated palmitoylated lipid A with an additional L-Ara4N moiety (m/z 915.60, exact mass 1831.20) were also observed, in relation with the increased expression of *pagP* and *arnT*. Two peaks, *bis*-phosphorylated penta-acylated lipid A with *m/z* 714.48 (exact mass 1428.95) and *bis*-phosphorylated hexa-acylated lipid A with *m/z* 939.65 (exact mass 1879.29) identified in WT strain, were not detected after the treatment with 3’,6-dinonyl neamine.

Exposure of ∆*mlaA* to 3’,6-dinonyl neamine produced no new peaks as compared to the *∆mlaA* but we observed a significantly higher abundance of a *mono*-phosphorylated tetra-acylated lipid A (*m/z* 1211.76), a *bis*-phosphorylated penta-acylated lipid A (*m/z* 722.39 exact mass 1444.78), a *bis*-phosphorylated palmitoylated with two additional L-Ara4N moieties lipid A (hexa-acylated) (*m/z* 951.57 exact mass 1903.15), and the disappearence of a *bis*-phosphorylated hexa-acylated lipid A (*m/z* 807.5 exact mass 1614.98). Exposure of ∆*mlaA* strain to 3’,6-dinonyl neamine also decreased the production of *mono*-phosphorylated penta-acylated lipid A at *m/z* 1337.86, which is in contrast with what was observed in WT treated strain.
